# Phytochemical Characterization and Neuropharmacological Assessment of *Combretum indicum* Methanolic Extract With Integrated Molecular Docking and Dynamics Simulation for Anxiety and Depression Therapy

**DOI:** 10.1002/fsn3.71918

**Published:** 2026-05-23

**Authors:** Zobayed Islam, Md. Ashraful Alam, Sakib Ahamed Bhuiyan, Sabikun Naher, Nilufar Sultana, S. M. Naim Uddin

**Affiliations:** ^1^ Department of Pharmacy, Faculty of Biological Science University of Chittagong Chattogram Bangladesh; ^2^ Department of Pharmacy Jashore University of Science and Technology Jashore Bangladesh; ^3^ Department of Pharmacy Manarat International University Dhaka Bangladesh

**Keywords:** anxiety, *Combretum indicum*, depression, ethnomedicine, neuropharmacology

## Abstract

A study of the phytochemical composition, as well as in vitro and in vivo neuropharmacological activities of methanol extract of *C. indicum* (*MECI*) was carried out along with an in silico prediction. GC–MS/MS and FTIR results showed that the PEs contained various bioactive components like flavonoids, terpenoids, alkaloids, heterocyclic substances. In mice, *MECI* (200 and 400 mg/kg, i.p.) caused significant dose‐dependent anxiolytic effects as indicated by the elevated plus maze test (increased duration and entries in open arms; 166.6 ± 14.71 s, ***p < 0.001), hole‐board test (enhanced head‐dipping frequency; 53.4 ± 4.71; ***p < 0.001), light/dark box test (greatest latency time without suppression of locomotion). Antidepressant‐like effect was evidenced by decreased immobility in forced swim (48.5%–55.1% reduction) and tail suspension test (83.0 ± 2.60 to 72.4 ± 2.33 s), being at the level of efficacy of fluoxetine (67.4 ± 2.87 s). The extract did not have a sedative effect as measured by the open matter and hole‐cross tests. ADME profiling suggested well oral absorption and BBB permeability and PASS prediction indicated possible neuropharmacological activity. Molecular docking revealed that the major constituents (methyl 10,13‐dimethyltetradecanoate; −8.5 kcal/mol) and tetrazolo[1,5‐b]pyridazine (−8.1 kcal/mol) showed strong binding scores with CNS targets as compared to reference drugs. Molecular dynamics simulation revealed that Tetrazolo[1,5‐b]pyridazine forms a structurally stable complex with the 2Z5X protein, supporting its potential anxiolytic activity through favorable conformational stabilization. Toxicity predictions suggested low hepatotoxicity and cardiotoxicity. These results in turn offer mechanistic explanation for ethnomedicinal use of *C. indicum* to manage anxiety and depression and thus endorse it as a multi‐target natural therapeutic agent against the two mood disorders.

## Graphical abstract

Graphical abstract illustrating the preparation of methanolic extract from the whole plant of *Combretum indicum* , followed by phytochemical characterization using GC–MS and FTIR analyses. The study integrates in vivo neuropharmacological investigations with in silico computational approaches, including molecular docking, ADMET profiling, PASS prediction, and drug‐likeness evaluation, to elucidate the mechanistic pathways underlying its anxiolytic, sedative, and antidepressant activities and to assess its therapeutic potential as a natural neuropharmacological agent.

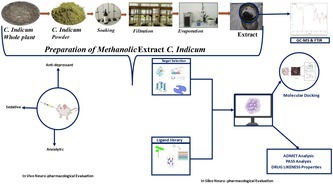

## 1 Introduction

Neuropsychiatric (NP) disorders like anxiety and depression are a significant global health burden, resulting in significant disability, poor quality of life, and socioeconomic loss globally, especially in low‐ to middle‐income countries with limited access to mental healthcare (Zhang et al. 2023). While the current pharmacotherapeutics such as monoaminergic agents and selective serotonin reuptake inhibitors are commonly in practice, a significant percentage of patients experience delayed therapeutic onset, incomplete remission, side effects, and high rates of relapse only when they discontinue treatment, while synthetic drug discovery still meets with high attrition. Outcomes such as these highlight the need for alternative and/or adjunctive approaches to CNS drug discovery (Siddiqui et al. 2022; Chaachouay and Zidane 2024). Medicinal plants represent a valuable source of structurally diverse bioactive compounds with significant neuropharmacological potential (Gonçalves et al. 2025; Chen et al. 2025). Their ability to modulate multiple CNS targets, coupled with comparatively lower toxicity profiles, makes them possible candidates for the development of safer and more effective drugs for neurological disorders. Moreover, plant‐derived phytochemicals offer a strategic advantage in discovering novel lead compounds for anxiolytic, antidepressant, and neuroprotective therapies (Gargiulo et al. 2026). Medicinal plants constitute a rich and ever‐expanding source of structurally diverse and chemically unique bioactive compounds, as over 25% of modern medicines are based on plant secondary metabolites or their derivatives while nearly 80% of the world's human population rely on herbal medicine for primary health care in developing countries. The worldwide herbal market is expected to cross $400 billion by 2030, indicating an increasing scientific and commercial interest in natural therapeutics. Bangladesh, by itself, has more than 500 medicinal plant species with high ethnopharmacological importance, some of which still have not been thoroughly investigated scientifically for their neuroactive potential (El‐Saadony et al. 2025). *Combretum indicum* (syn. *Quisqualis indica* L.), commonly employed in South and Southeast Asia to treat gastrointestinal, inflammatory, and nervous problems, includes flavonoids, triterpenoids, phenolics, alkaloids, and glycosides—classes known for anti‐anxiety & antidepressant activities (Abd Elkarim and Taie 2023; Neriyana and Alva 2020). Nevertheless, its neuropharmacological profile has not been extensively described. Thus, an attempt has been made to explore the phytochemical composition and neuropharmacological potential of methanol extract of *C. indicum* in a systematic way with the help of in vivo and in silico techniques in the present study.

Neuropsychiatric disorders, such as anxiety and depression, are now among the greatest contributors to worldwide disability burden for all ages (Yuan et al. 2023). Current pharmacotherapies—for example, benzodiazepines for anxiety and selective serotonin reuptake inhibitors (SSRIs) for depression—are clinically effective but often cause side effects such as sedation, cognitive impairment, tolerance, dependence, sexual dysfunction, and delayed onset of action. These restrictions have highlighted the need for safer, multi‐target phytotherapeutic agents that modulate central neurotransmitter systems with better tolerability (Nawrot et al. 2022; Remali and Aizat 2024). Notably, flavonoids and phenolic compounds, in general, show some binding capabilities for GABAergic and monoaminergic systems, providing a possible mechanistic rationale for the anxiolytic, sedative, and antidepressant activity from medicinal plants seen for these functions (Drevets et al. 2022; Dobrek and Głowacka 2023).

The novelty of the current work is to narrow the gap between folk use and pharmacological validation of *Combretum indicum* in CNS disorders (Kenda et al. 2022). Although neuroactive effects of some phytoconstituents from closely related species have been observed, much less has been known about the comprehensive neuropharmacological activities of the whole plant extract of *C. indicum* . Due to complex phytochemical synergy in crude plant extracts, the whole‐plant approach can demonstrate broader and more meaningful therapeutic profiles than isolated components alone. Based on this evidence, systematic behavioral and pharmacological screening of *C. indicum* might establish groundwork for bioassay‐guided fractionation, identification of lead compounds for neuropsychiatric drug discovery in the future (Abd Elkarim and Taie 2023; Forid et al. 2021; Md Abu Hanif et al. 2020).

Beyond in vivo behavioral pharmacology, modern neuropharmacological studies also increasingly incorporate molecular‐level analysis both to rationalize observed bioeffect to help speed hit identification (Rahman et al. 2023). Molecular docking allows for mechanistic elucidation of ligand–target interactions in terms of predicted binding affinities and patterns of interactions between the bioactive phytocomponents and some CNS‐related protein targets associated with anxiety, sedation, and depression. Also, pharmacokinetic and safety profiling in the early stages is crucial for assessing translational potential. In silico absorption, distribution, metabolism, and excretion (ADME) prediction and drug‐likeness evaluation provide an initial estimation of oral bioavailability, blood–brain barrier penetration, and metabolic stability. Activity Spectra for Substances (PASS) predictions offer a probabilistic insight into potential biological activities and computational toxicity profiling allow early estimation of adverse liabilities, such as hepatotoxicity, cardiotoxicity and mutagenicity. The incorporation of these computational filters with experimental neurobehavioral results provides a more comprehensive framework for prioritizing candidate neurotherapeutics (Davis and Choisy 2024; Hassan et al. 2023).

Molecular dynamics (MD) simulation is a powerful computational approach used to evaluate the stability and dynamic behavior of protein–ligand complexes at an atomic level over time. Prior to MD simulation, molecular docking is commonly employed to predict the binding orientation and affinity of ligands within the active site of target proteins. While docking provides a static snapshot of protein–ligand interactions, MD simulation offers deeper insights by capturing conformational flexibility, binding stability, and interaction persistence under dynamic conditions. In neuropharmacological studies, this combined approach plays a crucial role in validating the therapeutic potential of bioactive compounds. Therefore, this study integrated molecular docking and MD simulation to assess the binding and stability of the 2Z5X–Tetrazolo[1,5‐b]pyridazine complex derived from *Combretum indicum* (Azme et al. 2025; Mohapatra et al. 2023).

In the present study, we have evaluated the anxiolytic, sedative, and antidepressant effects of whole plant extract of *Combretum indicum* in a sequential manner using established in its vivo neurobehavioral models. It was proposed to determine anxiolytic potential involving modulation of anxiety‐related behavior and GABAergic signaling, sedative activity indicating central depressant properties and inhibitory neurotransmission, while antidepressant activity would indicate effects in behavioral despair paradigms needing monoaminergic regulation. These studies in combination with molecular docking, ADME profiling, drug‐likeness prediction, PASS, and toxicity screening would testify to the complete neuropharmacological standardization of *C. indicum* and provide a solid foundation to promote it as a natural lead molecule for further development against neuropsychiatric diseases.

## 2 Methods

### 2.1 Plant Collection, Identification, and Crude Extract Preparation

The whole plant of *Combretum indicum* (L.) DeFilipps was collected from Botanical Garden, University of Chittagong, Bangladesh and identified by a taxonomist in the Department of Botany, University of Chittagong. A voucher specimen was also made and deposited at the departmental herbarium for reference. The plant materials were washed with distilled water, shade‐dried at room temperature and grounded into powdered form (coarse). Fine powder of the plant material (800 g) was macerated in 100% Pure methanol for 15 days with shaking at intervals. The extract was filtered through muslin cloth and then with Whatman No. 1 filter paper, concentrated in vacuo using a rotary evaporator to obtain the crude extract. About 20 g of dried whole plant extract were collected and preserved in airtight container at 4°C for use in future pharmacological and phytochemical studies.

### 2.2 Preliminary Phytochemical Screening

Preliminary phytochemical analysis of the qualitative assay for secondary metabolites of methanolic extract of *Combretum indicum* (*MECI*) was performed. Test for the presence of major bioactive classes (polyphenols, flavonoids, tannins, alkaloids, saponins triterpenoids, and steroid) were carried using standardized phytochemical analysis. This initial chemotypic profile was aimed at furnishing a molecular basis for the following pharmacological screenings and to direct the isolated on‐demand natural products (Maheshwaran et al. 2024; Assiry et al. 2023).

### 2.3 Gas Chromatography–Mass Spectrometry (GC–MS/MS) Analysis

GC–MS/MS analysis of methanolic extract of *Combretum indicum* The GC–MS/MS analysis of the methanolic extract was carried out at the central analytical facility, Jashore Science and Technology University using a Shimadzu GCMS‐TQ 8040 system (Shimadzu Corporation, Kyoto, Japan) fitted with a DB‐5 ms fused‐silica capillary column (30 m × 0.25 mm i.d., film thickness 0.25 μm). Helium (99.999% purity) was used as carrier gas at a flow rate of 1.0 mL/min, with injection of 1 μL of the sample and splitless mode.

The injector was operated at 280°C. The temperature program of the oven was as follows: 60°C (0 min), heating until constant from 60°C to 240°C at a rate of 4°C/min, and then maintaining the temperature for 15 min, resulting in a total run time of about < 52 min. The mass spectrometer was set in electron ionization (EI) mode, 70 eV. The ion source and interface temperatures were set as recommended by the manufacturer (Teoh et al. 2023; Üst et al. 2024). Tentative phytoconstituents and their possible identification were directly based on matching of the obtained mass spectra with those of the National Institute of Standards and Technology library supplemented with a comparison to retention index when available. Only high spectral similarity score compounds were included in the tentative annotation.

### 2.4 Fourier Transform Infrared (FTIR) Spectroscopic Analysis

The characteristic functional groups present in the ethanolic extract of *Combretum indicum* were identified using Fourier Transform Infrared (FTIR) spectroscopy. FTIR spectrum of the sample was obtained in the mid‐infrared region (4000–400 cm^−1^) and processed for systematic interpretation for assignment of diagnostic absorption bands to known functional groups which also corroborate different major classes of phytoconstituents. FTIR analysis was conducted with the PerkinElmer Spectrum Two FTIR spectrophotometer (central analytical facility of Jashore Science and Technology University, Jashore, Bangladesh). Measurement of the sample was done in solid state with the KBr pellet technique. The spectral range was 4000–400 cm^−1^ with a resolution of 4 cm^−1^ and 16 co‐added scans were performed to improve the signal‐to‐noise ratio. The baseline corrected and cross‐referenced characteristic absorption frequencies of the recorded spectrum were compared to standard functional group correlation charts and reported literature data (Pasieczna‐Patkowska et al. 2025; Ulpathakumbura et al. 2023).

### 2.5 In Vivo Evaluation of Anxiolytic Activity in Mice

#### 2.5.1 Elevated Plus Maze Test

Investigation on esterified extract of *C. indicum* was done to study the purported anxiolytic effects using elevated plus maze (EPM) model of anxiety‐like behavior. The apparatus consisted of two opposite open arms (50 × 10 cm) and two opposite closed arms (50 × 10 × 40 cm), connected by a central platform (10 × 10 cm) that was raised to a height of 50 cm from the floor. Mice were gently placed on the central square facing one open arm at the start of each trial and they were allowed to explore the maze freely for 5 min. Open arm and closed arm entries were counted as the indices of anxiety‐related exploratory behavior and risk assessment. All behavioral measurements were performed in a constant, background ambient environment with minimal extraneous alterations to sensory input, such that the height and open‐arm exposure of the maze constituted the main anxiety‐provoking stimuli. The EPM was wiped thoroughly with a 10% ethanol solution between trials to remove any retained olfactory cues and avoid inter‐subject behavioral contamination (Azad et al. 2025; Risdiana et al. 2025).

#### 2.5.2 Hole Board Test

Neurobehavioral effects of *Combretum indicum* extract using hole board model were observed to assess the exploratory behavior and neophilia induced anxiety responses. Apparatus The behavioral assessment was conducted in a wooden arena (40 × 40 × 25 cm) that had sixteen evenly spaced circular apertures (3 cm diameter) positioned throughout the floor, and raised 25 cm from the ground. Mice were put into the arena one by one at the beginning of each session and allowed to freely explore the chamber for 10 min. Frequency of line crossings (locomotor activity), head dipping behavior (exploratory drive and anxiolysis related index), and rearing (vertical exploratory drive) were measured as behavioral indices of anxiety‐like, exploratory phenotype. All test environments were maintained at constant environmental conditions with minimum possible extraneous sensory interference and disturbance; thus, responses were based on the inherent anxiogenic and exploratory nature of the apparatus. To avoid olfactory interference and interanimal behavioral carryover, the hole‐board platform was cleaned with a 10% ethanol solution before each trial (Arafat et al. 2025; Ali et al. 2025).

#### 2.5.3 Light Dark Test

Light/dark box was employed to investigate the potential anxiolytic effects of *Combretum indicum* extract on anxiety‐like behaviors. The apparatus consisted of two adjacent plastic boxes (20 × 10 × 14 cm) that are of the same size: a bright illuminated chamber (white walls) and a dark chamber (black walls), joined by an opened doorway allowing free bidirectional passage. The dark chamber was lit at 50 lx, the light compartment at 1000 lx (with an extra light source) to create a strong aversive contrast. At the beginning of each trial, mice were introduced in a gentle manner into the light compartment facing the connecting alley and were free to explore both compartments for 5 min. The transitions between compartments and the total time inside the light chamber were measured as an index of anxiety‐like behavior and risk–avoidance characteristics. All experiments were carried out in tightly controlled environmental conditions with minimal extraneous sensory perturbation, such that differential illumination was the primary anxiogenic provocation. The experimental set‐up was cleaned with 10% ethanol solution between trials to eliminate residual olfactory cues and inter‐animal behavioral carryover (ScienceDirect Topics 2026b).

### 2.6 In Vivo Sedative Activity in Mice

#### 2.6.1 Open Field Test

The open‐field test was used to assess the anxiolytic‐like activity of *Combretum indicum* methanolic extract (MECI) based on anxiety‐associated behavioral parameters. The apparatus consisted of a wooden arena (96 × 96 × 30 cm) containing 16 squares (central grid, *n* = 4; peripheral grid, *n* = 12). Mice were placed in the corner and left to explore for 5 min. Behavioral outcomes were squares crossed (locomotion), rearings (vertical exploration) and grooming (stress‐coping). The apparatus was wiped between trials with a wet tissue and spirit. *MECI* (200, 400 mg/kg, I.p) was injected 30 min before the test. Experiments were performed in a stimulus‐reduced environment to eliminate or reduce any external cues, allowing us to determine if behavioral responses related to modulation due *MECI* (ScienceDirect Topics 2026c; Seibenhener and Wooten 2015).

#### 2.6.2 Hole Cross Test

Effect of *MECI* was determined on locomotor activity and sedative‐like effect by hole cross paradigm. The apparatus comprised a cage (30 × 20 × 14 cm) divided by a non‐removable partition with an aperture of 3 cm. One mouse at a time, which had been given either vehicle or *MECI*, was put into one side of the box and allowed to explore the hole leading to the other side of the chamber. Behaviors were observed during a 3‐min observation period before, and at 30, 60, 90, and 120 min after the treatment. This temporal recording allowed for dose‐dependent modulation of locomotor activity to be evaluated. The apparatus was thoroughly cleaned between trials and olfactory cues were removed to guarantee recorded behavior was due to *MECI*‐achieved neuromodulatory influence and not environmental bias (Ali et al. 2015; Shahed‐Al‐Mahmud and Lina 2017).

### 2.7 In Vivo Antidepressant Activity Test in Mice

The forced swim test (FST) is a well‐established and widely accepted experimental model for assessing behavioral despair and screening antidepressant activity (Trunnell et al. 2024; Dhangar et al. 2025). In the present study, the antidepressant potential of the methanolic extract of *Combretum indicum* (MECI) was evaluated using this validated paradigm. Mice were released into a cylinder of water (8 cm depth, 25°C) from an initial height of 20 cm. Immobility was described as absence of limb movement, apart from minimal movements necessary to maintain the head above the water (lifesaving response). The test was conducted over 2 days, with a 15‐min pre‐swim on the first day for stress adaptation and a 6‐min swim on the second day after deducting the first and last 2 min. After 4 min, immobility time was recorded to assess *MECI*‐induced antidepressant‐like behavior (Can, Dao, Arad, et al. 2011; ScienceDirect Topics 2026a).

#### 2.7.1 Tail Suspension Test

The antidepressant‐like activity of the methanolic extract of *Combretum indicum* (MECI) was assessed using the tail suspension test (TST), a robust and extensively validated model for behavioral despair. (Ali and Engidawork 2022; Serefko et al. 2025; Abebe et al. 2024). Mice were acoustically and visually isolated and suspended by adhesive tape under the belly (1 cm distance from the tip of their tail) at 50 cm over the floor. The mice were administered with *MECI* at 200 or 400 mg/kg (i.p.) and fluoxetine (20 mg/kg, i.p.) as a positive control. Freezing behavior was recorded for 4 min after a 2‐min habituation phase. Decreases in immobility versus control were considered to be an indication of an antidepressant‐like effect, that is, increased stress‐coping or active behavior (Can, Dao, Terrillion, et al. 2011).

### 2.8 Experimental Animals, Model Justification, Ethical Approval, and Behavioral Protocol

Swiss albino mice were selected for this study due to their suitability in neuropharmacological and behavioral research, including their genetic uniformity, well‐characterized physiology, and predictable responses in validated behavioral paradigms. These mice are widely employed in preclinical studies evaluating anxiolytic, sedative, and antidepressant effects, ensuring reproducibility with previous reports (Hua et al. 2026; Shamsi et al. 2025).

Animals were acclimatized to standard housing conditions for 7 days prior to experimentation (temperature 22°C ± 2°C; relative humidity 50%–60%; 12 h light/dark cycle). All behavioral tests were conducted during the light phase under controlled illumination (100–150 lx). To minimize order or habituation effects, tests were conducted in a standardized sequence from most stressful to least stressful (open field test → elevated plus maze → hole‐board test → forced swim test), with at least 24 h between each test. Observations and data recording were performed by trained personnel blinded to treatment groups to limit experimental bias (Russell et al. 2021; Singha et al. 2020).

All procedures complied with internationally accepted standards for the care and use of laboratory animals, aiming to minimize suffering. The study was approved by the Animal Research Ethics Committee of the Faculty of Biological Sciences, University of Chittagong, Bangladesh (Approval No. AERB‐FBSCU‐20260220, February 20, 2025). Sample sizes (*n* = 5 per group) were determined based on previous studies and in accordance with the 3Rs principles (Reduction, Replacement, Refinement) to balance statistical reliability with ethical considerations.

Special arrangements included the use of anesthesia for any invasive procedures and predefined humane endpoints to minimize pain or distress. At the end of the study, animals were humanely euthanized via overdose of a suitable anesthetic (e.g., ketamine/xylazine), and death was confirmed before proper disposal in accordance with institutional guidelines (Kooliyattayil et al. 2025).

### 2.9 Acute Oral Toxicity Study

In accordance with Organization for Economic Cooperation and Development (OECD) Guidelines (Acute Toxic Class Method) to check its security, the toxic biological test was conducted on mice The test extract was given orally to 2000 mg per kilo of body weight by limited dose, and the control group received an equal volume of the liquid vehicle Here for the initial 4 h after administration, animals were observed closely at all times and then at various intervals throughout a 24‐h period That was followed by more frequent observations over a period of 2 weeks as a measure to detect delayed toxicity and mortality. Observations included changes in skin and fur, eyes, mucous membranes, as well respiratory patterns such as how fast air came from the nostrils or how deeply it was drawn into lungs; autonomic and central nervous system activity; behavioral patterns on drugs or odors, tremors in sleep; convulsions; salivation which might be due to vomiting or excessive thirst accompanied by a dry mouth; diarrhea indeed both caused for different reasons—diarrhea alongside other symptoms presently mentioned ought not be taken away at this time simply because they have happened earlier; lethargy; sleepiness; Sleep; no death occurred and no signs of toxicity greater than those typical conspecifics encountered with LD 50 has been detected within the tested dose range of RM at all… Even though it might seem hard to believe, these results tended to suggest a certain degree of safety. Thus three test doses were chosen: 200, 400, and 500 mg/kg body weight, respectively (OECD 2001; Hafiz Mail et al. 2025; Saleem et al. 2017).

### 2.10 In Silico Activity

#### 2.10.1 Drug Likeness Properties

Drug‐likeness profile of *Combretum indicum* phytoconstituents was analyzed with SwissADME (https://www.swissadme.ch/) and ADMETlab 3.0. The determinants evaluated were molecular weight, number of hydrogen‐bond acceptor and donor groups, skin permeability (logKp, cm/s), solubility, gastrointestinal absorption, Lupinski and Veber rule violations, bioavailability score as well as PAINS alerts.

#### 2.10.2 PASS Prediction

Pharmacological activities and mechanisms of action of the *Combretum indicum* phytoconstituents were predicted using PASS (Prediction of Activity Spectra for Substances) Online method to predict potential therapeutic biomedical uses based on structure–activity relationships.

#### 2.10.3 ADME Analysis

The pharmacokinetic (absorption, distribution, metabolism and excretion/toxicity) properties were determined in silico (in vitro or in vivo) ADME property via SwissADME (https://www.swissadme.ch/) for *Combretum indicum* phytoconstituents. The specific features were water solubility (logmol/L), human intestinal absorption (%), volume of distribution at steady state (VDss, logL/kg), blood–brain barrier permeability (logBB), status as a CYP3A4 substrate, total clearance (logmL/min/kg), Ames mutagenicity and hepatotoxicity.

#### 2.10.4 Ligand Selection, Targets and Molecular Docking, Visualization

A total of 31 phytoconstituents identified through GC–MS analysis of *Combretum indicum* methanolic extract were sketched using ChemDraw and cross‐validated with the PubChem database. Ligands were standardized using Open Babel to ensure consistent protonation states and proper charge assignment.

**FIGURE 1:**
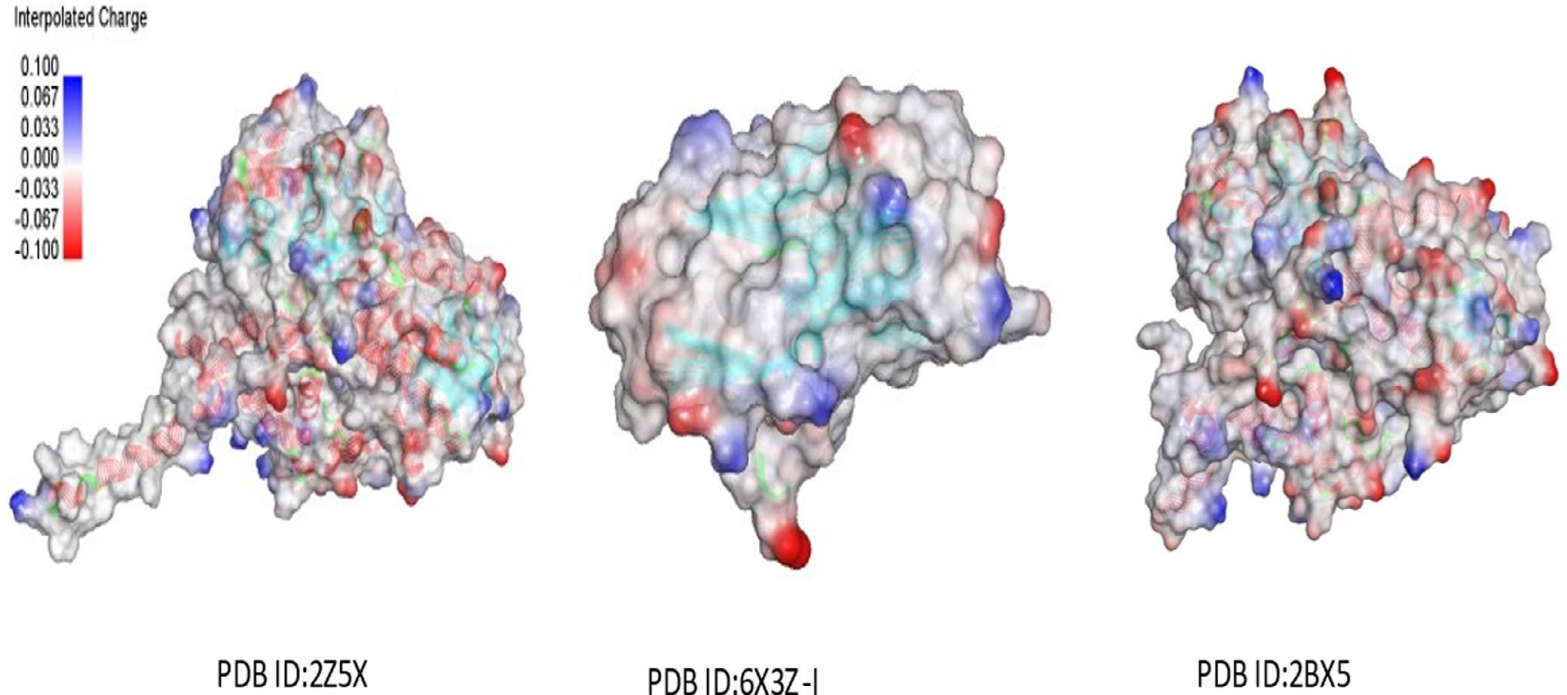
In silico molecular docking analysis of target proteins associated with anxiolytic, sedative, and antidepressant activities.

High‐resolution crystallographic structures were obtained from the RCSB Protein Data Bank for three neuropharmacologically relevent targets including Monoamino oxidase‐A inhibition (PDB ID: 2Z5X) (Ansari et al. 2025), γ‐aminobutyric acid type A (GABA‐A) receptor (PDB ID: 6X3X) (Kim et al. 2020) and serotonin receptor in complex with its inhibitor (PDB ID: 2BX5) (Abebe et al. 2024), based on their known involvement in anxiolytic, sedative or antidepressant pathways as shown in Figure 1. The structure was pre‐processed by Discovery Studio Visualizer deleting excess heteroatoms and PDB waters, and by energy minimization with SWISS‐PDBViwer for the removal of excessive steric clashes and optimization of side chain conformations. Molecular docking was performed using AutoDock Vina within MGLTools. Polar hydrogens were added, Kollman charges were assigned to the receptors, and Gasteiger charges were applied to the ligands prior to docking. The grid box parameters were defined based on the coordinates of co‐crystallized ligands to accurately encompass the active binding pockets. For MAO‐A (PDB ID: 2Z5X), the grid center was set at (*x* = 40.12, *y* = 12.55, *z* = 28.37) with dimensions of 22 × 22 × 22 Å. For the GABAA_AA receptor (PDB ID: 6X3X), the grid center was defined at (*x* = −15.34, *y* = 18.62, *z* = 72.41) with a box size of 24 × 24 × 24 Å. For the serotonin receptor (PDB ID: 2BX5), the grid center was set at (*x* = 30.78, *y* = −2.91, *z* = 14.66) with dimensions of 22 × 22 × 22 Å. These parameters were optimized to cover both orthosteric and adjacent allosteric regions, enabling adequate conformational sampling. Docking was performed with an exhaustiveness value of 8, generating up to 9 binding poses per ligand, and an energy range of 3 kcal/mol. Reference ligands were selected based on established activity against their respective targets to ensure biologically relevant comparisons. Binding affinities and interaction profiles were analyzed; however, the results were interpreted as supportive evidence rather than definitive proof of pharmacological activity. Visualization using PyMOL revealed stabilizing interactions such as hydrogen bonding and π–π stacking, which may contribute to ligand binding. Docking outcomes were cautiously correlated with behavioral findings, without implying direct mechanistic causation.

#### 2.10.5 Molecular Dynamic Simulation

Using Molecular Dynamics (MD) Simulations to Investigate the Structural Stability and Dynamic Behavior of the Protein‐Ligand Complex Composed Binding Target Protein (PDB ID: 2Z5X) and Tetrazolo [1,5‐ b] removing Dihydrates from *Combretum indicum* . The protein structure was preprocessed by removing crystallographic water molecules, adding missing hydrogen atoms, and performing energy minimization to eliminate steric clashes. The GROMACS force field was applied to the protein, while ligand topology parameters were generated using the PRODRG server. The protein–ligand complexes were then placed in a cubic simulation box with a minimum distance of 1.0 nm from the box edges and solvated using the TIP3P water model. Counter ions were subsequently added to neutralize the system.

Energy minimization was performed using the steepest descent algorithm with a convergence threshold of 1000 kJ/mol/nm to remove unfavorable van der Waals and electrostatic interactions. The system was then equilibrated in two phases: an NVT ensemble for 100 ps at 300 K using the V‐rescale thermostat to maintain constant number of particles, volume, and temperature, followed by an NPT ensemble for 100 ps at 1 bar using the Parrinello–Rahman barostat to maintain constant pressure. With a cut‐off distance of 1.0 nm for Coulombic and van der Waals interactions in each case, all the other parts of the periodic boundary condition were implemented. In addition, particle mesh Ewald electrosystem C kinetic interactions calculated long‐range electrostatic interactions. The LINCS algorithm was employed to constrain all bond lengths. The product MD simulation ran for 100 ns with a time step of 2 fs, and trajectory data was collected every 10 ps. Finally, GROMACS tools were used to analyze the parameters of key structures, that is, Root mean squared deviation (RMSD) Root mean squared fluctuation (RMSF) Radius of gyration (Rg.). These results were used to evaluate the conformational stability and compactness of the protein–ligand complex throughout the course of the simulation (Shoaib et al. 2023; Gazmeh et al. 2024; El Khatabi et al. 2022).

#### 2.10.6 Toxicity Analysis

In silico toxicity profiling of *C. indicum* phytoconstituents was carried out by ProTox‐3 (https://tox.charite.de/protox3/) to know the LD_50_ values and assign them to different classes of toxicity assessing the hepatotoxicity, cardiotoxicity, carcinogenicity, cytotoxicity, and neurotoxicity.

### 2.11 Statistical Analysis

Data from all experiments are shown as mean ± SEM with *n* = 5. Statistical analysis was performed with SPSS. One‐way analysis of variance (ANOVA) was conducted, and Dunnett's post hoc test was applied for inter‐group comparisons. *p* < 0.05, *p < 0.01, and **p < 0.001 were considered statistically significant compared to the control group. All vitro assays were performed in triplicate, and graphical representations were generated using Microsoft Excel, PowerPoint, and GraphPad Prism.

## 3 Result

### 3.1 Phytochemical Screening

Preliminary phytochemical screening of methanol extract of *Combretum indicum* (*MECI*) was positive for various bioactive compounds. Result of phytochemical screening showed the presence of alkaloids, cardiac glycosides, flavonoids, phenolic compounds, tannins, and saponins thus presenting a wide array of (Figure 1) phytoconstituents (Table 1). These molecules have various pharmacological activities indicating *MECI* might hold great therapeutic effect. The uniform occurrence of these secondary metabolites indicates that the extract is chemically complex and traditional in medicine.

**TABLE 1:** Preliminary phytochemical screening of the methanol extract of *Combretum indicum* .

SL. no.	Phytochemicals	Test	Inference
1	Alkaloids	(a) Dragendroff's test	+
		(b) Hager's test	+
		(c) Mayer's test	−
		(d) Wagner's test	−
2	Reducing sugars	Fehling's test	−
3	Glycosides	(a) 10% NaOH test	−
		(b) Aqueous NaOH test	−
4	Cardiac glycosides	(a) Keller‐Killani test	+
		(b) Bromine water test	+
5	Flavonoids	(a) Alkaline reagent test	+
		(b) Ammonia test	−
		(c) Conc. Sulfuric acid test	+
6	Phenolic compounds	(a) Iodine test	+
		(b) Ferric chloride test	−
		(c) Lead acetate test	+
7	Tannins	(a) 10% NaOH test	+
		(b) Braymer's test	−
8	Saponins	(a) Foam test	++
		(b) NaHCO_3_ test	++
9	Terpenoids	Chloroform test	+
10	Quinones	Conc. HCl test	_
11	Carboxylic acid	Effervescence test	−
12	Resins	Acetic Anhydride test	+

*Note:* Semi‐quantitative biological activity was expressed using a (+) scale, where − indicates no activity, + weak activity, ++ moderate activity, +++ strong activity, and ++++ very strong activity. The in vitro anthelmintic assay was performed in triplicate.

### 3.2 GCMS Profiling of Methanolic Extract of *C. indicum*

**FIGURE 2:**
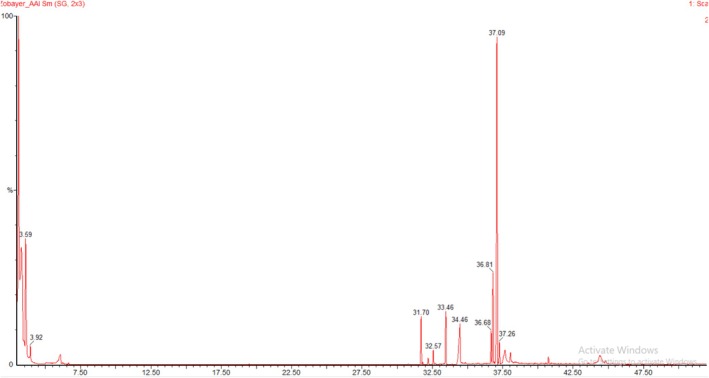
Total ion chromatogram (TIC) of the methanolic whole plant extract of *Combretum indicum* *obt*ained by GC–MS/MS analysis, showing the distribution of volatile and semi‐volatile phytoconstituents across the retention time window.

The methanolic extract of *C. indicum* (*MECI*) has been shown to contain chemically diverse phytocomponents which were heterocyclic nitrogenous, aliphatic, hydrocarbons, oxygenated terpenoids, fatty acids and their esters profiles by gas chromatography–mass spectrometry (GC–MS/MS). The TIC representative of the four ATLO samples is depicted in at Figure 2 with reasonably well‐resolved peaks spanning the whole acquisition time. The tentative identification of the individual constituents was performed according to their retention times (RT) relative to a *n*‐alkane series, mass spectra with the NIST library, molecular weights and relative peak area percentages and they are presented in Table 2.

**TABLE 2:** GC–MS/MS‐identified phytoconstituents of the methanolic extract of *Combretum indicum* with their retention times (RT), molecular weights, and relative peak area percentages.

Serial no.	Retention time (RT)	Name of the compounds	Molecular weight	%Area
1	3.09	Tetrazolo[1, 5‐b]pyridazine	121	0.01
2	3.28	Ethane, 2‐chloro‐1‐ethoxy‐1‐methoxy‐	138	10.73
3	3.59	5‐Nitro‐2‐aminobenzophenone thiosemicarbazone	315	9.24
4	6.04	1,3,7‐Octatrien‐5‐yne	104	2.72
5	6.63	2‐Amino‐5‐methylbenzoic acid	151	0.11
6	19.47	1,3‐Dioxolane, 2‐(dichloromethyl)‐	156	0.03
7	30.79	2,3,4‐Trimethylpentanoic acid	144	0.04
8	31.70	Neophytadiene	278	3.56
9	31.82	3‐Methyl‐2‐(2‐oxopropyl)furan	138	0.13
10	32.20	Cyclohexene,3‐propyl‐	124	0.45
11	32.57	Cis, cis‐1,9‐dimethylspiro[5.5]undecane	180	1.00
12	33.46	Tetradecanoic acid, 10,13‐dimethyl‐, methyl ester	270	4.12
13	34.46	12‐Bromododecanoic acid	278	7.24
14	34.84	Pentanoic acid, 4‐methyl‐	116	0.16
15	35.39	(R)‐(−)‐4‐Methylhexanoic acid	130	0.08
16	36.68	Methyl 12,13‐tetradecadienoate	238	2.45
17	36.81	Z, Z, Z‐8, 9‐epoxyeicosa‐5, 11, 14‐trienoic acid, methyl ester	334	6.97
18	37.09	Phytol	296	29.13
19	37.26	Heptacosanoic acid, 25‐methyl‐, methyl ester	438	1.59
20	37.66	2‐Fluoro‐7‐hydroxybicyclo [2.2.1] heptane	130	3.44
21	38.05	N‐Decanoic acid	172	1.10
22	40.22	2,4,4‐Trimethyl‐1‐pentanol	130	0.02
23	40.75	Heptacosanoic acid, 25‐methyl‐, methyl ester	438	0.58
24	43.59	1,3‐Dioxolane, 2‐butyl‐2‐ethyl‐	138	0.01
26	44.41	Dodecane, 1‐fluoro‐	188	3.76
27	44.80	2‐Pentanone, 1,3‐dimethoxy‐3‐methyl‐	160	0.64
28	45.29	Formic acid, neopentyl ester	116	0.17
29	47.18	1‐Propanone, 1‐[2‐(1,1‐dimethylethyl)cyclopropyl]‐2,2‐dimethyl‐, trans‐	182	0.10
30	47.63	2‐N‐butylthiolane, S,S‐dioxide	176	0.04
31	47.98	4‐octanol, 7‐methyl‐	144	0.05

All of the identified components, some of pharmacological interest were identified as neophytadiene (RT 31.70 min), phytol (RT 37.09 min), long‐chain fatty acid derivatives (e.g., methyl 12,13‐tetradecadienoate; Z,Z,Z‐8,9‐epoxyeicosa‐5,11,14‐trienoic acid methyl ester) and oxygenate hydrocarbons. The existence of diterpenoid alcohols (phytol), unsaturated fatty acids and their methyl esters, also substituted heterocyclic rings indicates that a chemically diverse matrix may collectively account for the antioxidant, antimicrobial, cytotoxic and wound‐healing activities exhibited by the *MECI*. This phytochemical diversity of *C. indicum* suggests that the observed bioactivities may arise from the combined and complementary effects of multiple low‐molecular‐weight constituents present in the extract, rather than being attributable to a single dominant compound.

### 3.3 FTIR Analysis of *Combretum indicum* Extract

**FIGURE 3:**
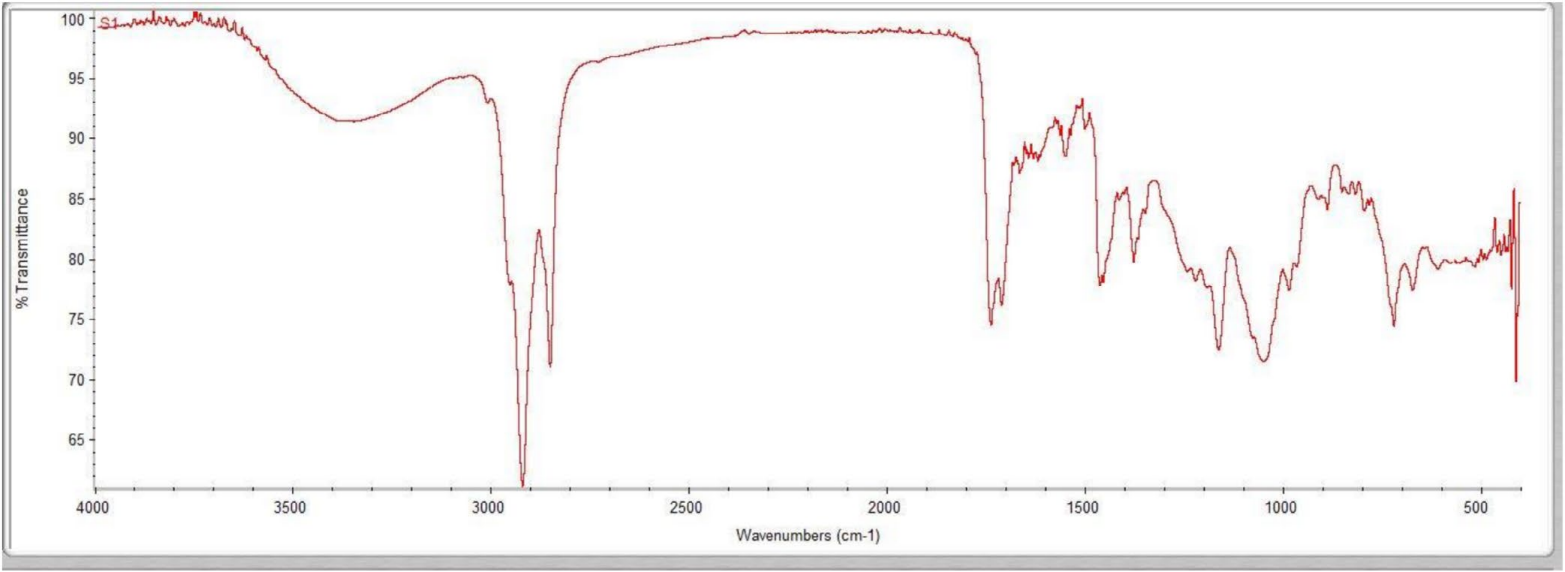
Identification of functional groups in *MECI* by FTIR analysis.

FTIR analysis revealed the functional group composition of the methanolic extract of *Combretum indicum* and gave insights into its various phytochemical constituents at molecular level. The FTIR spectrum (Figure 3) displayed a set of resolved vibrational bands associated with different types of chemical bonds existing in the extract and allowed tentative assignment of compound classes shown in Table 3.

**TABLE 3:** FTIR absorption peaks and their corresponding functional group assignments for *Combretum indicum* extract.

Peak region (cm^−1^)	Functional group	Compound class interpretation
3400–3200	O–H stretching	Alcohols, phenols, hydrogen‐bonded compounds
2920–2850	C–H stretching	Alkanes, fatty acids, terpenoids
1736–1710	C=O stretching	Esters, ketones, aldehydes
1600–1500	C=C stretching/N–H bending	Aromatic rings, amides
1450–1370	C–H bending	Aliphatic hydrocarbons
1250–1050	C–O stretching	Ethers, esters, alcohols
800–600	C–H out‐of‐plane bending	Aromatic substitution patterns

The relatively strong wide absorption band in the range of 3400–3200 cm^−1^ is present which can be attributed to O–H stretching vibration corresponding to alcohols, phenol and H‐bonded molecules. The bands between 2920 and 2850 cm^−1^ are related to CH stretching due to aliphatic hydrocarbons, like fatty acids and terpenoids. The heavy absorption at 1736–1710 cm^−1^ corresponds to C=O stretching, indicating the presence of esters, ketones and aldehydes. The peaks at 1600–1500 cm^−1^ can be attributed to C=C stretching and N–H bending of aromatic ring and amide groups. The other bands at 1450–1370 cm^−1^ (asymmetrical stretching mode of C–H) and at [1250–1050 cm^−1^] (stretching mode of C–O with oxygenated functional groups, esters, ethers and alcohols) are also detected which supports the collection of aliphatic hydrocarbons and oxygen‐containing compounds like alcohol, ether as well as ester. In the last place, the bands in the 800–600 cm^−1^ region are assigned to C–H out of plane bending and are representative of substitution patterns on the aromatic ring.

Overall, the FTIR fingerprint spectrum confirms the presence of diverse functional groups in *C. indicum* extract, including hydroxyl (O–H), carbonyl (C=O), and aromatic (C=C) moieties, indicating a chemically rich phytochemical profile. This functional group‐level characterization supports the GC–MS‐based identification of bioactive constituents and provides a structural basis for the observed pharmacological activities, including antioxidant, antimicrobial, and wound‐healing potential.

### 3.4 Evaluation of In Vivo Anxiolytic Activity

#### 3.4.1 Evaluation of Anxiolytic‐Like Activity Using the Elevated Plus Maze

**FIGURE 4:**
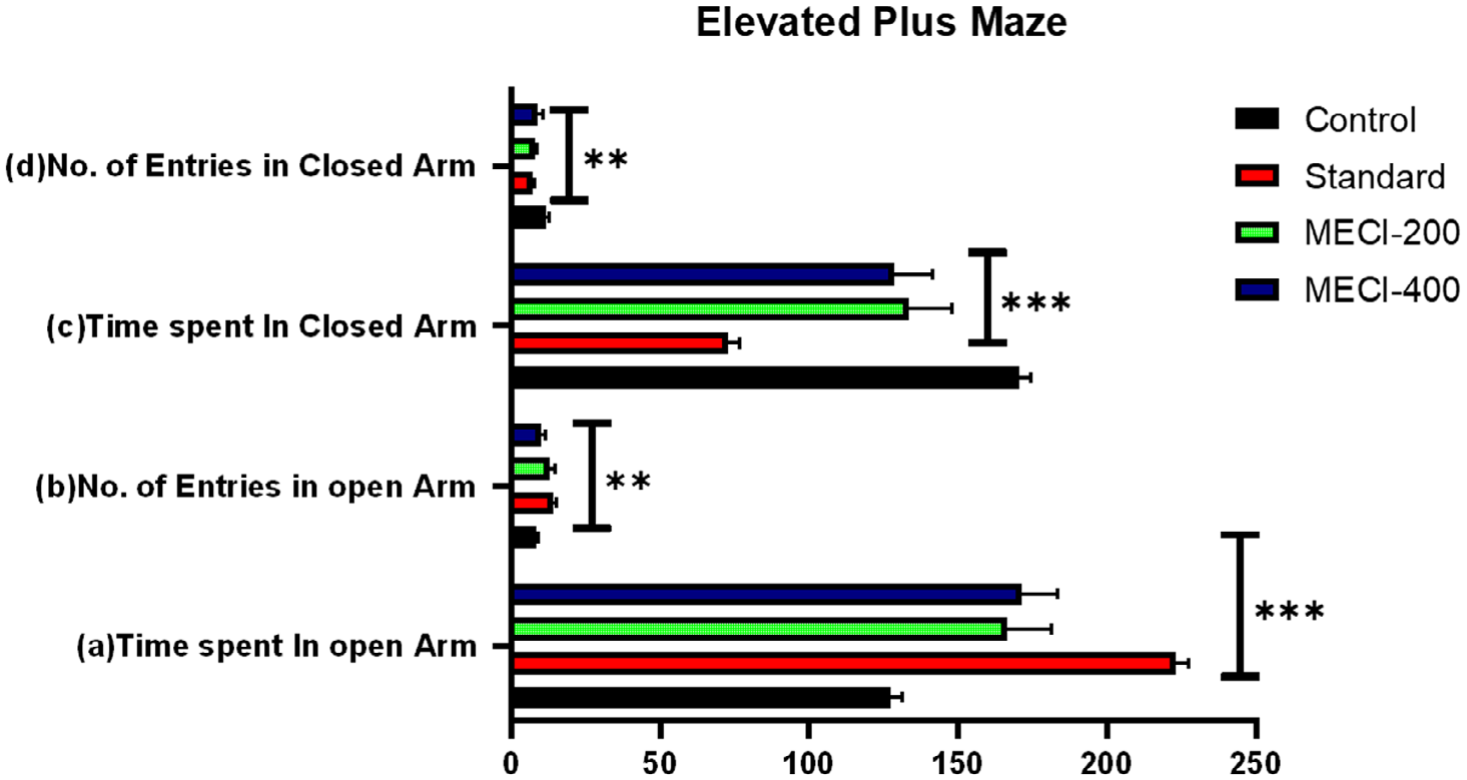
Effects of *Combretum indicum* methanolic extract (*MECI*) on exploratory behavior in the elevated plus maze. Bar graphs depict (a) time spent in open arms, (b) number of open‐arm entries, (c) time spent in closed arms, and (d) number of closed‐arm entries following treatment with *MECI* (200 and 400 mg/kg) and standard anxiolytic drug. Data are presented as mean ± SEM. Statistical significance relative to control is indicated as **p < 0.01 and ***p < 0.001.

Effect of *Combretum indicum* methanolic extract (*MECI*) on anxiety‐like behavior in mice was by pretension (Table 4, Figure 4). When compared with control, the traditional anxiolytic therapy led to a robust increase of both the duration in open arms (223.2 ± 4.12 s), thus validating the model experimental sensitivity. The results obtained in this study indicate that *MECI* treatment at doses of 200 and 400 mg/kg produced a significant anxiolytic‐like behavioral profile (Table 4). *MECI* (200 mg/kg) significantly increased open‐arm exploration as demonstrated by more time spent in the open arms (166.6 ± 14.71 s, ***p < 0.001) along with the higher number of entries into the open arms (12.8 ± 1.72, ***p < 0.001), coupled with a decrease in closed arm occupancy (133.4 ± 14.71 s, **p < 0.01) and closed arm visits (7.8 ± 0.74, **p < 0.01). Additionally, *MECI* at 400 mg/kg conspicuously extended time on the open arms (171.4 ± 12.19 s, ***p < 0.001) and shortened time spent in the closed arms (128.6 ± 12.91 s, ***p < 0.001). Changes in open‐arm entries (10.0 ± 1.41) or closed arm entries (8.6 ± 1.85) did not achieve statistical significance, but the direction of effects implied a profile indicative of an anxiolytic‐like effect overall (Figure 4). Altogether, these results suggest that MECI induces dose‐dependent anxiolytic‐like effects in the EPM test, as evidenced by increased exploration of the aversive open arms and reduced preference for the enclosed arms, thereby confirming the neurobehavioral effect of phytoconstituents from *Combretum indicum* . Consistent with these findings, the light/dark box test was further employed to validate and extend the observed anxiolytic‐like activity across an additional behavioral paradigm.

**TABLE 4:** Effects of *Combretum indicum* methanolic extract (*MECI*) on anxiety‐related parameters in the elevated plus maze test.

Group	Time spent in open‐arm (mean ± SEM)	No. of entries in open‐arm (mean ± SEM)	Time spent in closed arm (Mean ± SEM)	No. of entries in closed‐arm (Mean ± SEM)
Control	127.4 ± 3.79	8.4 ± 0.51	170.6 ± 3.91	11.6 ± 0.93
Standard	223.2 ± 4.12***	14.2 ± 1.02**	72.6 ± 4.08***	7.2 ± 0.58**
*MECI*‐200	166.6 ± 14.71***	12.8 ± 1.72**	133.4 ± 14.71***	7.8 ± 0.74**
*MECI*‐400	171.4 ± 12.19***	10 ± 1.41**	128.6 ± 12.91***	8.6 ± 1.85

*Note:* Each value represents the mean ± SEM, (*n* = 5). One‐way ANOVA followed by Dunnett's test. Symbols in this section indicate statistical significance relative to the control group: *p < 0.05; **p < 0.01; ***p < 0.001.

#### 3.4.2 Evaluation of Anxiolytic‐Like Activity Using the Hole‐Board Test

**FIGURE 5:**
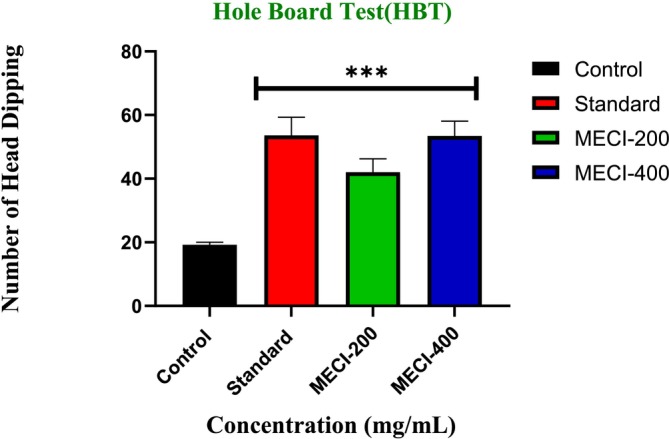
The Hole Board Test was employed to examine the anxiolytic properties of *MECI*. Bar plots represent the mean number of head dips observed during the test session following administration of vehicle, diazepam (1 mg/kg), and *MECI* (200 and 400 mg/kg). Data are presented as mean ± SEM. Statistical significance versus control is denoted as ***p < 0.001.

The effects of behavior and novel responses were observed through the hole‐board paradigm to determine the anxiolytic‐like activity of *C. indicum* methanolic extract (*MECI*) (Table 5, Figure 5). The vehicle‐treated control (1% Tween 80) group displayed a significantly lower frequency of head‐dipping behavior (19.2 ± 0.86), indicating a basal neophobic response in the novel environment. By contrast, the referent anxiolytic, diazepam (1 mg/kg), produced a strong and significant increase in head‐dip frequency (53.6 ± 5.67; ***p < 0.001) confirming the predictive sensitivity of hole‐board testing to detect anxiolytics. In particular, the number of head dips was markedly higher (42.0 ± 4.24, ***p < 0.001) following *MECI* treatment at a dose of 200 mg/kg when compared with control values demonstrating alleviation of anxiety‐like behavior as well as augmented interest to explore the novel apertures for mice treated with *MECI* compound. In addition, at a dose of 400 mg/kg *MECI* significantly pronounced the head‐dipping response (53.4 ± 4.71; ***p < 0.001) and matched that induced by administration of diazepam alone (Table 5). This marked increase in head‐dipping counts represents a powerful anxiolytic‐like action and more exploratory behavior. Taken together, these findings indicate that MECI significantly enhanced exploratory and risk‐assessment behaviors in the hole‐board test in mice, suggesting that *Combretum indicum* contains bioactive constituents capable of modulating anxiety‐related neurocircuitry. Notably, the greater magnitude of effect observed at the higher dose supports a clear dose–response relationship in behavioral efficacy, comparable to that of a classical benzodiazepine anxiolytic (Figure 5). To further strengthen the translational relevance and confirm the consistency of these anxiolytic‐like effects, additional behavioral paradigms in mice were subsequently employed.

**TABLE 5:** Evaluation of anxiolytic activity of *MECI*.

Test sample	Dose (mg/kg)	Number of head dipping					Mean ± SEM
		M1	M2	M3	M4	M5	
1% Tween 80	0.10 mL/10 g	17	19	22	18	20	19.2 ± 0.86
Diazepam	1	50	49	60	61	48	53.6 ± 5.67***
*MECI*	200	48	41	44	35	42	42 ± 4.24***
*MECI*	400	46	54	51	60	56	53.4 ± 4.71***

*Note:* Each value represents the mean ± SEM, (*n* = 5). One‐way ANOVA followed by Dunnett's test. Symbols in this section indicate statistical significance relative to the control group: *p < 0.05; **p < 0.01; ***p < 0.001.

#### 3.4.3 Evaluation of Anxiolytic‐Like Activity Using the Light–Dark Box Test

**FIGURE 6:**
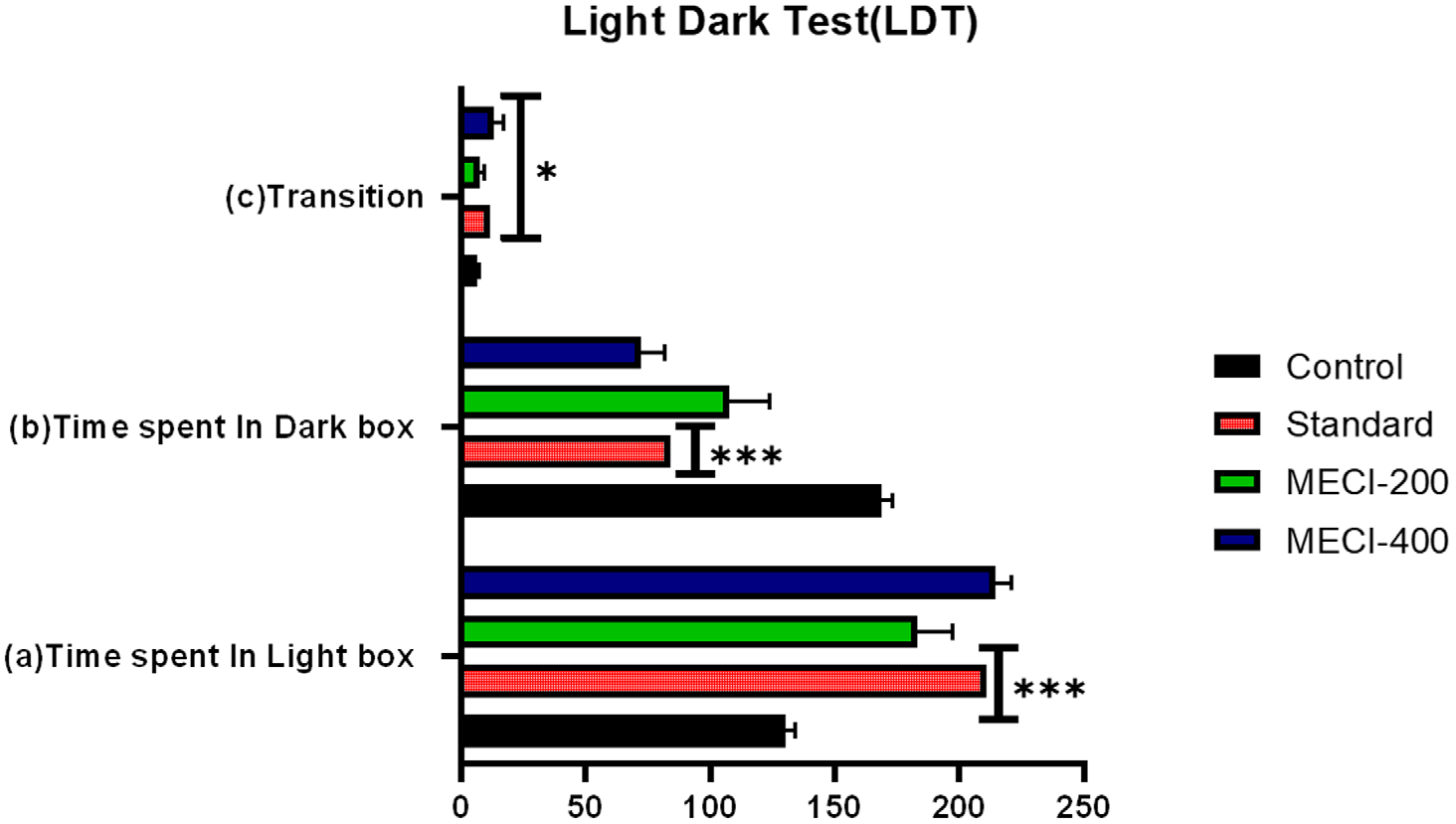
Evaluation of anxiolytic activity through light–dark box test. Bar graphs illustrate (a) time spent in the light compartment, (b) time spent in the dark compartment, and (c) number of transitions between compartments following treatment with vehicle, diazepam (1 mg/kg), and *MECI* (200 and 400 mg/kg). Data are presented as mean ± SEM. Statistical significance relative to control is denoted as *p < 0.05 and ***p < 0.001.

To further substantiate the anxiolytic‐like potential of *Combretum indicum* (Table 6, Figure 6), the light/dark box test was employed as a well‐established behavioral model for evaluating anxiety‐related responses in mice. In this assay, vehicle‐treated control mice demonstrated a marked aversion to the illuminated compartment, spending significantly less time in the light chamber (130.4 ± 3.85 s) compared to the dark chamber (168.8 ± 4.35 s). This behavioral pattern reflects the innate anxiety‐like tendency of mice to avoid brightly lit and open spaces when exposed to a novel and potentially threatening environment. Consistent with this, time spent in the light compartment was significantly increased by the reference anxiolytic diazepam (211.2 ± 3.98 s, ***p < 0.001) with an accompanying decrease in dark‐box occupancy (84.4 ± 3.72 s, ***p < 0.001), and increase in transitions between compartments (11.8 ± 0.97, *p < 0.05), which confirm predictive validity of the assay. Treatment with *MECI* dose dependently reduced the anxiety‐like behavior. MCIE of 200 mg/kg had a significant enhancement in the light‐box residence time (183.2 ± 14.23 s) and reduction in the dark‐compartment time (107.6 ± 16.18 s), although it failed to reach a statistical difference from the control group. Conversely, *MECI* at 400 mg/kg markedly extended the time of light‐box exploration (214.8 ± 6.40 s) and significantly reduced dark‐box entry (72.2 ± 9.50 s), along with significant increase in transfers between compartments (13.0 ± 4.04), collectively demonstrating a potent anxiolytic‐like behavioral profile that is comparable to standard anxiolytic (Table 6). Collectively, our observations confirm the anti‐anxiety–like activity of *MECI* in the light/dark box and complement previous converging behavioral evidence from elevated plus maze and hole‐board standardized assays, thereby advocating for the neuropharmacological action *of Combretum indicum*–derived phytoconstituents.

**TABLE 6:** Evaluation of anxiolytic activity of *MECI* through Light–Dark Box Test.

Group	Time spent in light box (s) (mean ± SEM)	Time spent in dark box (s) (mean ± SEM)	Transitions (mean ± SEM)
Control	130.4 ± 3.85	168.8 ± 4.35	6.6 ± 0.81
Standard	211.2 ± 3.98***	84.4 ± 3.72***	11.8 ± 0.97*
*MECI*‐200	183.2 ± 14.23	107.6 ± 16.18	7.4 ± 2.15*
*MECI*‐400	214.8 ± 6.4	72.2 ± 9.5	13 ± 4.04*

*Note:* Each value represents the mean ± SEM, (*n* = 5). One‐way ANOVA followed by Dunnett's test. Symbols in this section indicate statistical significance relative to the control group: *p < 0.05; **p < 0.01; ***p < 0.001.

### 3.5 Evaluation of In Vivo Sedative Activity

#### 3.5.1 Evaluation of Locomotor Activity Using the Open Field Test

**FIGURE 7:**
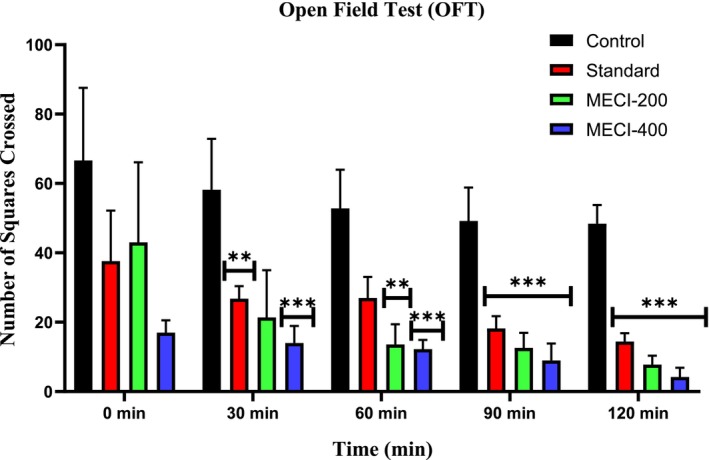
The Open Field Test was employed to examine the sedative properties of *MECI*.

Locomotor activity of methanolic extract of *Combretum indicum* (*MECI*) consumption‐induced effect on locomotor activity was assessed using open field test in mice and the results are presented in Table 7 and Figure 7, respectively. As shown in Table 7, animals of the control group performed a high number at 0 min (66.6 ± 20.94), which decreased gradually at all time points but still relatively higher through the observation periods. On the contrary, the standard group anesthetized with diazepam at 1 mg/kg significantly inhibited behavioral patterns of locomotion in all intervals and this could verify its anticipated CNS depressing effect. *MECI* (200 mg/kg) significantly and time‐dependently decreased the number of movements. Such decreases met statistical significance at 30 min and were observed for each of the remaining time points (**p < 0.01 to ***p < 0.001) (Table 7). Greater suppression of spontaneous motor activity in ME‐400 mg/kg‐treated groups was observed at all time intervals as compared to the ME‐200 mg/kg treated group, and was quite significant (***p < 0.001) with respect to the negative control group. These effects can be seen in Figure 7, where both *MECI*‐200 and *MECI*‐400 treated groups show a dose‐dependent reduction of exploratory activity as a function of time that closely resemble the activity generated after administration of diazepam.

**TABLE 7:** Comparative effect of *MECI* and standard drug on open field test performance.

Animal Group	Number of movement at 0 min	Number of movement at 30 min	Number of movement at 60 min	Number of movement at 90 min	Number of movement at 120 min
Control	66.6 ± 20.94	58.2 ± 14.66	52.8 ± 11.14	49.2 ± 9.6	48.4 ± 5.4
Standard	37.6 ± 14.55	26.8 ± 3.6	27 ± 6.06	18.2 ± 3.54	14.4 ± 2.41
ME‐200	43 ± 23.12	21.4 ± 13.58**	13.6 ± 5.78***	12.6 ± 4.36***	7.8 ± 2.56***
ME‐400	17 ± 3.54***	14 ± 4.93***	12.2 ± 2.71***	9 ± 4.85***	4.2 ± 2.71***

*Note:* All values are shown as mean ± SEM, and statistical analysis is performed using One‐Way Analysis of Variance (ANOVA). Subsequently, *n* = 5 is employed for Dunnett's multiple comparison test, with *p < 0.05, **p < 0.01, and ***p < 0.001 in comparison to the control group.

#### 3.5.2 Hole Cross Test

**FIGURE 8:**
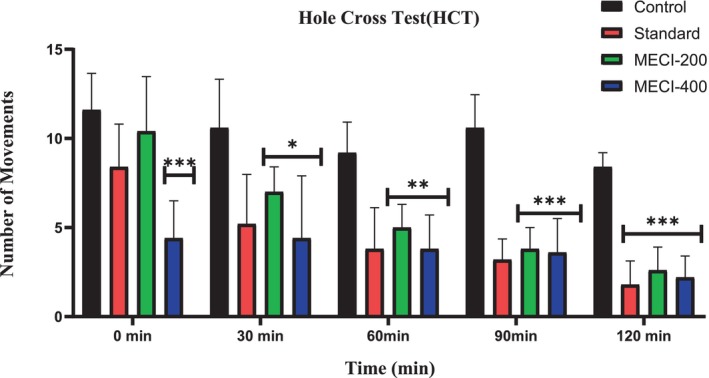
The Hole Cross Test was employed to examine the sedative properties of *MECI*.

In the hole‐cross model, both methanolic extract from *Combretum indicum* ‐treated groups were not able to show significant reduction in spontaneous locomotion throughout the observation period than vehicle‐treated control group (Table 8; Figure 8). By comparison, the positive control sedative diazepam significantly reduced locomotor transitions at 30 min post injection as confirmation of the sensitivity of the assay to CNS depressants. As reported before, a definite reduction in the number of hole crossings compared to the control and standard drug‐treated rats suggests sedative or CNS‐depressant action that may exhibit dose‐dependent suppression of locomotor activity which is more pronounced at higher concentrations of extract. In particular, *MECI* did not produce a significant decrease in locomotor parameters at the doses examined, nor was it sedative liability. Taken together, these results indicate that the anxiolytic‐like behavioral action of *C. indicum* seen in various ethological paradigms (EPM, hole‐board and LDT) are least likely to be hindered by non‐specific motor retardation or sedation, supporting a specific anxiolytic profile without marked locomotor function disruption.

**TABLE 8:** Comparative sedative effect of *MECI* and Diazepam in the Hole Cross Test.

Animal group	Number of movements				
	0 min	30 min	60 min	90 min	120 min
Control	11.6 ± 2.05	10.6 ± 2.72	9.2 ± 1.72	10.6 ± 1.85	8.4 ± 0.8
Standard	8.4 ± 2.41	5.2 ± 2.78	3.8 ± 2.31	3.2 ± 1.16	1.8 ± 1.32***
ME‐200	10.4 ± 3.07	7 ± 1.41*	5 ± 1.26**	3.8 ± 1.16***	2.6 ± 1.32***
ME‐400	4.4 ± 2.05***	4.4 ± 3.49*	3.6 ± 1.85**	3.6 ± 1.85	2.2 ± 1.16***

*Note:* Data are expressed as mean ± SEM. *p < 0.05, **p < 0.01, ***p < 0.001 were considered statistically significant.

### 3.6 Evaluation of In Vivo Antidepressant Activity

#### 3.6.1 Evaluation of Antidepressant‐Like Activity Using the Forced Swimming Test

**FIGURE 9:**
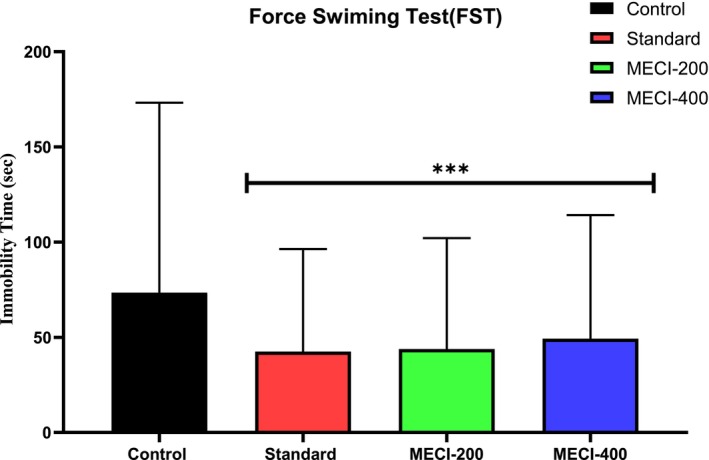
The Forced Swimming Test was employed to examine the antidepressant properties of *MECI*.

Forced Swim Test (FST) FST is a common behavior screen, which offers the advantage of being designed as an easy and rapid method for the selection of substances with more antidepressant activity. Treatment with *MECI* at 200 and 400 mg/kg significantly and dose‐dependently decreased the immobility time compared to the vehicle‐treated control group (Table 9, Figure 9). This reduction of behavioral despair is a measure of enforcement of an antidepressant‐like profile. Rodent exposed to uncontrollable noxious environment predictably shift progressively to immobility, as a sign of behavioral despair and stress‐coping failure. In this study, *MECI*‐treated animals also showed a continued increase in active coping behaviors represented by a consistent reduction of immobility time, which indicated a prolonged stress‐resilient mood phenotype and motivational persistence. The magnitude of effect at the higher dose also favors a dose‐dependent antidepressant‐like action of *MECI*. Taken as a whole, these results indicate that *Combretum indicum* exhibits potent antidepressant‐like activity in the FST, thereby supporting its neuropharmacological prospect and extending the anxiolytic‐like profile evidenced by its actions in the elevated plus maze, hole‐board, and light/dark box tests.

**TABLE 9:** Evaluation of antidepressant activity through Forced Swimming Test.

Sample	Dose (mg/kg)	Immobility time						% Immobility
		S‐1	S‐2	S‐3	S‐4	S‐5	Mean ± SEM	
Control	10	140	137	154	142	147	144 ± 2.98	
Fluoxetine	1	84	73	87	91	68	80.6 ± 4.34***	44.03
*MECI*	200	81	87	86	83	88	85 ± 2.60***	40.97
*MECI*	400	99	93	95	99	90	95.2 ± 3.48***	33.88

*Note:* All values are expressed as mean ± SEM, and statistical analysis is performed using One‐Way Analysis of Variance (ANOVA). Next, *n* = 5 is used for Dunnett's multiple comparison test, with *p < 0.05, **p < 0.01, and ***p < 0.001 when compared to the control group.

#### 3.6.2 Evaluation of Antidepressant‐Like Activity Using the Tail Suspension Test

**FIGURE 10:**
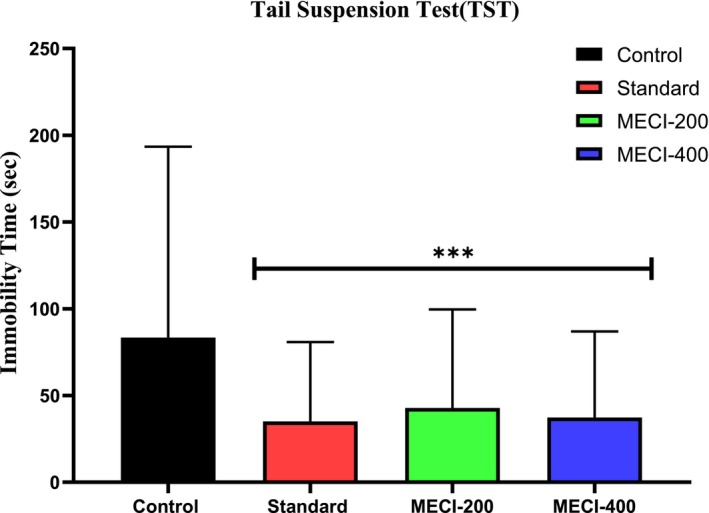
The Tail Suspension Test was employed to examine the antidepressant properties of *MECI*. Bar graphs depict immobility time in seconds following treatment with vehicle, fluoxetine (10 mg/kg), and *MECI* (200 and 400 mg/kg). Data are presented as mean ± SEM. Statistical significance relative to control is indicated as ***p < 0.001.

Additionally the antidepressant property of *MECI* was studied using tail suspension test (TST) which is a validated model for depression in rodents. Control ones (vehicle‐treated) were characterized by the prolonged period of immobility (161.2 ± 5.67 s), which demonstrates the baseline of depressive‐like behavior in Table 10 and Figure 10. Fluoxetine (10 mg/kg) used as reference antidepressant significantly decreased the immobility time to 67.4 ± 2.87 s (*p* < 0.001), which represented a decrease of 58.2% in immobilization, confirming the predictive sensitivity of TST behavior test. Interesting, *MECI* treatment dose‐dependently induced a marked reduction in immobility time. *MECI* administered at 200 mg/kg decreased immobility time to 83.0 ± 2.60 s (*p* < 0.001, reduction of 48.5%) and the higher dose of 400 mg/kg attenuated immobility further to reach a value of 72.4 ± 2.33 s (***p < 0.001), reduction of for fluoxetine (54:45%). The decrement in immobility observed here might be owing to *MECI*'s facilitating some form of active stress‐coping strategies during the inescapable event, and yielding a diminishment of behavioral despair. These results support the antidepressant‐like effects of *C. indicum* and reinforce its efficiency also in the forced test against despair, suggesting that some of the bioactive chemical components from its extract have a modulatory action on neurobehavioral stress adaptation and mood behavior.

**TABLE 10:** Evaluation of antidepressant activity through Tail Suspension Test.

Sample	Dose (mg/kg)	Immobility time						% Immobility
		S‐1	S‐2	S‐3	S‐4	S‐5	Mean ± SEM	
Control	10	168	145	178	154	161	161.2 ± 5.67	—
Fluoxetine	10	76	65	59	71	66	67.4 ± 2.87***	58.19
*MECI*	200	84	85	86	81	79	83 ± 2.60***	48.51
*MECI*	400	76	71	73	69	73	72.4 ± 2.33***	55.08

*Note:* All values are expressed as Mean ± SEM, and statistical analysis is performed using One‐Way Analysis of Variance (ANOVA). Next, *n* = 5 is used for Dunnett's multiple comparison test, with *p < 0.05, **p < 0.01, and ***p < 0.001 when compared to the control group.

### 3.7 In Silico

#### 3.7.1 Drug Likeness Properties

A computational in silico analysis of important ADME descriptors was executed to assess the pharmacokinetic capability of the major phytoconstituents confirmed in *Combretum indicum* , as given in Table 11. The tested compounds (Tetrazolo[1,5‐b]pyridazine, 3‐Methyl‐2‐(2‐oxopropyl)furan, Cyclohexene, 3‐propyl‐, Tetradecanoic acid, 10,13‐dimethyl‐methyl ester, and Methyl 12,13‐tetradecadienoate) showed molecular weights less than 500 Da, fulfilling virtually drug‐likeness cut‐offs.

**TABLE 11:** Drug‐likeness and pharmacokinetic properties of selected *MECI* phytochemicals.

Compound name	Pubchem ID	MW	H‐bond acceptors	H‐bond donors	log Kp (cm/s)	Solubility	GI absorption	Lipinski #violations	Veber #violations	Bioavailability Score	PAINS #alerts
Tetrazolo[1, 5‐b]pyridazine	548600	121.1	4	0	−7.12	Very soluble	High	0	0	0.55	0
3‐Methyl‐2‐(2‐oxopropyl)furan	545772	138.16	2	0	−6.14	Very soluble	High	0	0	0.55	0
Cyclohexene,3‐propyl‐	138092	110.2	0	0	−4.61	Soluble	Low	0	0	0.55	0
Tetradecanoic acid, 10,13‐dimethyl‐, methyl ester	175468	279.21	2	1	−4.43	Moderately soluble	High	0	1	0.85	0
Methyl 12,13‐tetradecadienoate	91692385	238.37	2	0	−4.37	Soluble	High	0	1	0.55	0

H‐bond donor/acceptor numbers were at acceptable levels, and Tetrazolo[1,5‐b]pyridazine was determined to carry the greatest acceptor density (*n* = 4) while maintaining good permeability indexes. Predicted water solubility varied from very soluble to moderately soluble, indicating favorable dissolution kinetics. All log Kp were negative, which implied low permeability of the transdermal flux, and gastro‐intestinal absorption was mostly high, especially with lower molecular weight heterocycles scaffolds.

All compounds in this series met Lipinski's Rule of Five, with only the long alkyl ester derivatives processing a single Veber abnormality possibly due to improved conformational flexibility. However, the bioavailability factors (0.55–0.85) were in a reasonable range. Of note, no PAINS alerts were observed indicating low likelihood of assay interference.

Together, such data reinforce the possible drug‐like nature of the *C. indicum* phytoconstituents and confirm their potential to pass as structurally accessible lead scaffolds for further pharmacological exploitation.

#### 3.7.2 PASS Prediction

In silico biological activity spectra of prominent phytoconstituents of *C. indicum* were screened using PASS analysis (Table 12) and found to display overlapping neuropharmacological profiles in agreement with in vivo behavioral data. All screened compounds showed good Pa values for anxiolytic, antidepressant, and sedative activity along with low Pi suggesting high bioactivity probabilities. 3‐Methyl‐2‐(2‐oxopropyl)furan and cyclohexene‐3‐propyl had relatively higher Pa values for anxiolytic action and sedative effect; tetradecanoic acid, 10,13‐dimethyl‐, methyl ester was also found to exhibit a marked antidepressant‐like activity as well as sedative activity. Tetrazolo[1,5‐b]pyridazine had a well‐rounded multi‐modal neuroactivity action proposed to reflect pleiotropic CNS involvement. Combined, the increased Pa/Pi ratios in all functional domains suggest a polypharmacological activity of the *C. indicum* ingredients, confirming their mechanistic association to the observed anxiolytic, sedative, and antidepressant‐like phenotypes (40) and prioritizing these compounds for target‐oriented validation and lead optimization.

**TABLE 12:** Predicted biological activities of selected *MECI* phytochemicals using PASS software.

Compound name	Biological activity					
	Anxiolytic		Antidepressant		Sedative	
	Pa	Pi	Pa	Pi	Pa	Pi
Tetrazolo[1, 5‐b]pyridazine	0.65	0.12	0.69	0.53	0.76	0.13
3‐Methyl‐2‐(2‐oxopropyl)furan	0.92	0.03	0.65	0.45	0.87	0.24
Cyclohexene,3‐propyl‐	0.65	0.35	0.76	0.34	0.94	0.12
Tetradecanoic acid, 10,13‐dimethyl‐, methyl ester	0.76	0.45	0.89	0.32	0.97	0.21
Methyl 12,13‐tetradecadienoate	0.82	0.53	0.78	0.23	0.65	0.32

#### 3.7.3 ADME Analysis

The pharmacokinetic feasibility of the principal bioactive metabolites identified from *Combretum indicum* was predicted using in silico ADME profiling (Table 13). The hit compounds showed acceptable predicted human intestinal absorption indicating a good candidate for oral uptake, despite their varying aqueous solubility and lower solubility of tetrazolo[1,5‐b]pyridazine which can moderately restrict bioavailability. Predicted VDss values reflected moderate to high tissue distribution, particularly of lipophilic compounds, assuming sufficient systemic exposure. Importantly, all hit compounds had enough blood–brain barrier permeability (log BB > 0), therefore good brain penetration properties and a high candidate to approach central nervous system compartments in line with their neuropharmacological effects. Metabolic stability predictions indicated from low to moderate interaction with CYP3A4, and tetrazolo[1,5‐b]pyridazine was poorly prone for substrate tendency, indicative of low susceptibility for a rapid I‐phase metabolism and a relatively stable systemic exposure. Predicted values for total clearance fell within expectations, with long chain aliphatic derivatives showing relatively low clearances and presumably extended systemic half‐life. Overall, these ADME parameters suggest that the oral bioavailability of *C. indicum* ‐derived constituents is acceptable along with CNS distribution, manageable metabolic stability, and clearance profiles.

**TABLE 13:** Predicted ADME (absorption, distribution, metabolism, and excretion) properties of selected *MECI* phytochemicals.

Compounds name	Absorption		Distribution		Metabolism	Excretion	Toxicity	
	Water solubility (log mol/L)	Intestinal absorption (human; % Absorbed)	VDss (human) (log L/kg)	BBB permeability (log BB)	CYP3A4 substrate	Total clearance (log mL/min/kg)	AMES toxicity	Hepatotoxicity
Tetrazolo[1, 5‐b]pyridazine	0.025286	−4.66681	−0.14168	0.691518	1.66E‐05	5.535182	0.511889	0.49345
3‐Methyl‐2‐(2‐oxopropyl)furan	1.273639	−4.59885	−0.02938	0.785851	0.024682	10.00349	0.440473	0.168772
Cyclohexene,3‐propyl‐	3.609813	−4.78003	0.139155	0.881796	0.469103	9.21661	0.062252	0.166865
Tetradecanoic acid, 10,13‐dimethyl‐, methyl ester	6.40293	−5.0371	0.294759	0.04903	0.824031	6.691573	0.164256	0.341389
Methyl 12,13‐tetradecadienoate	4.772008	−5.01887	−0.14609	0.009579	0.858265	5.482279	0.326292	0.360822

#### 3.7.4 In Silico Computational Studies: Molecular Docking and Drug‐Likeness Assessment

Molecular docking studies of the major constituents of *MECI* against CNS‐related targets revealed significant binding affinities across both PyRx and CB‐Dock platforms. Several compounds, including PIDs 138092 and 548600, exhibited strong interactions with sedative, anxiolytic, and antidepressant targets, with docking scores ranging from −7.2 to −9.0 kcal/mol, comparable to reference drugs such as Diazepam, Lorazepam, and Methotrexate. These results indicate possible activity of *MECI* constituents, supporting the observed in vivo anxiolytic and antidepressant effects. The detailed docking scores of all tested compounds are presented in Table S1, which highlights the comparative binding affinities across different CNS targets and docking platforms. Overall, the in silico findings provide a mechanistic basis for the neuropharmacological efficacy of *Combretum indicum* and identify possible candidates for further CNS drug development.

#### 3.7.5 In Silico Evaluation of Anxiolytic Activity

**FIGURE 11:**
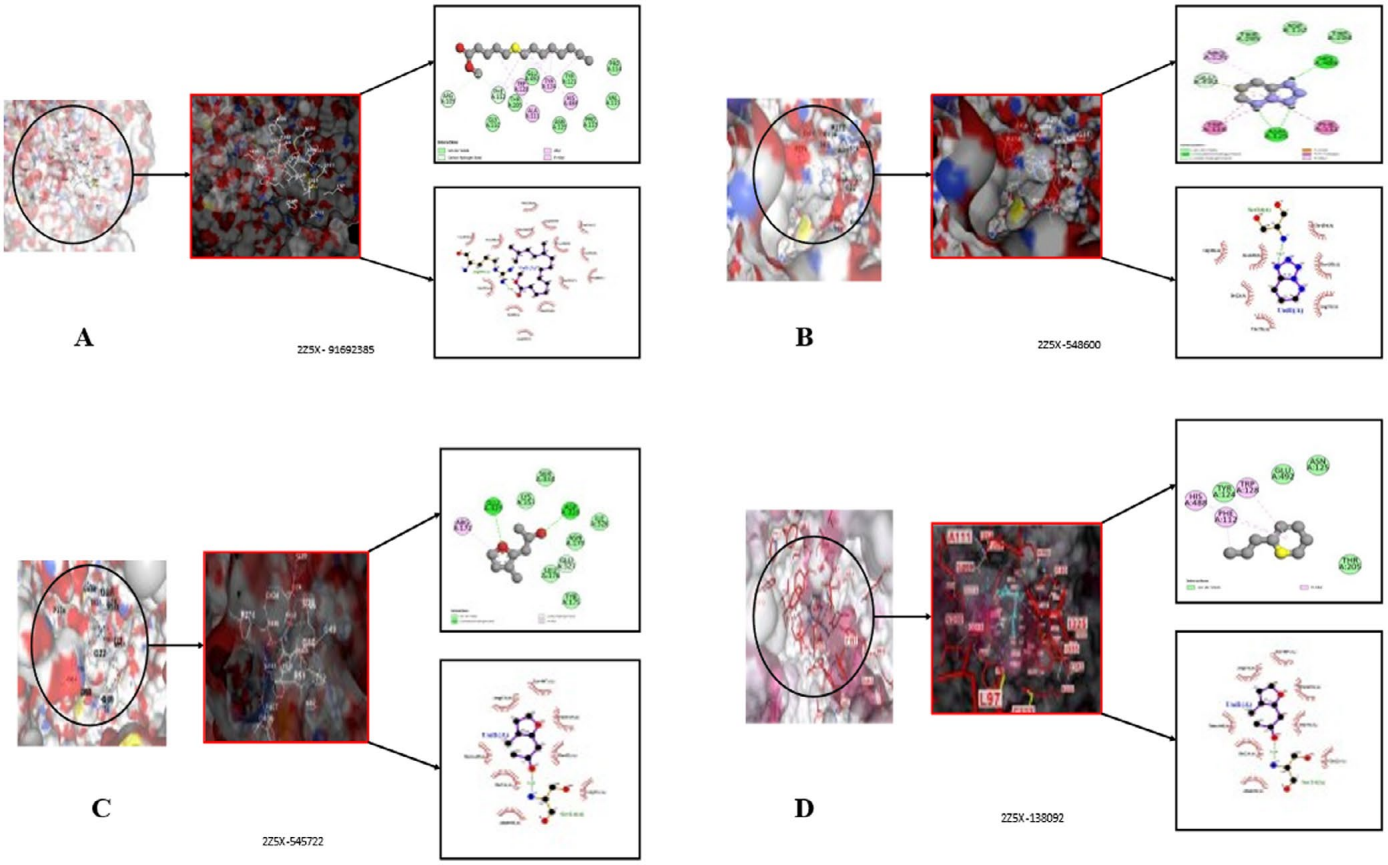
(A–D) Active‐site engagement profiles of selected phytoconstituents—(A) Methyl 12,13‐tetradecadienoate, (B) Tetrazolo[1,5‐b]pyridazine, (C) 3‐Methyl‐2‐(2‐oxopropyl)furan, and (D) Cyclohexene, 3‐propyl—within the anxiolytic target protein 2Z5X.

**FIGURE 12:**
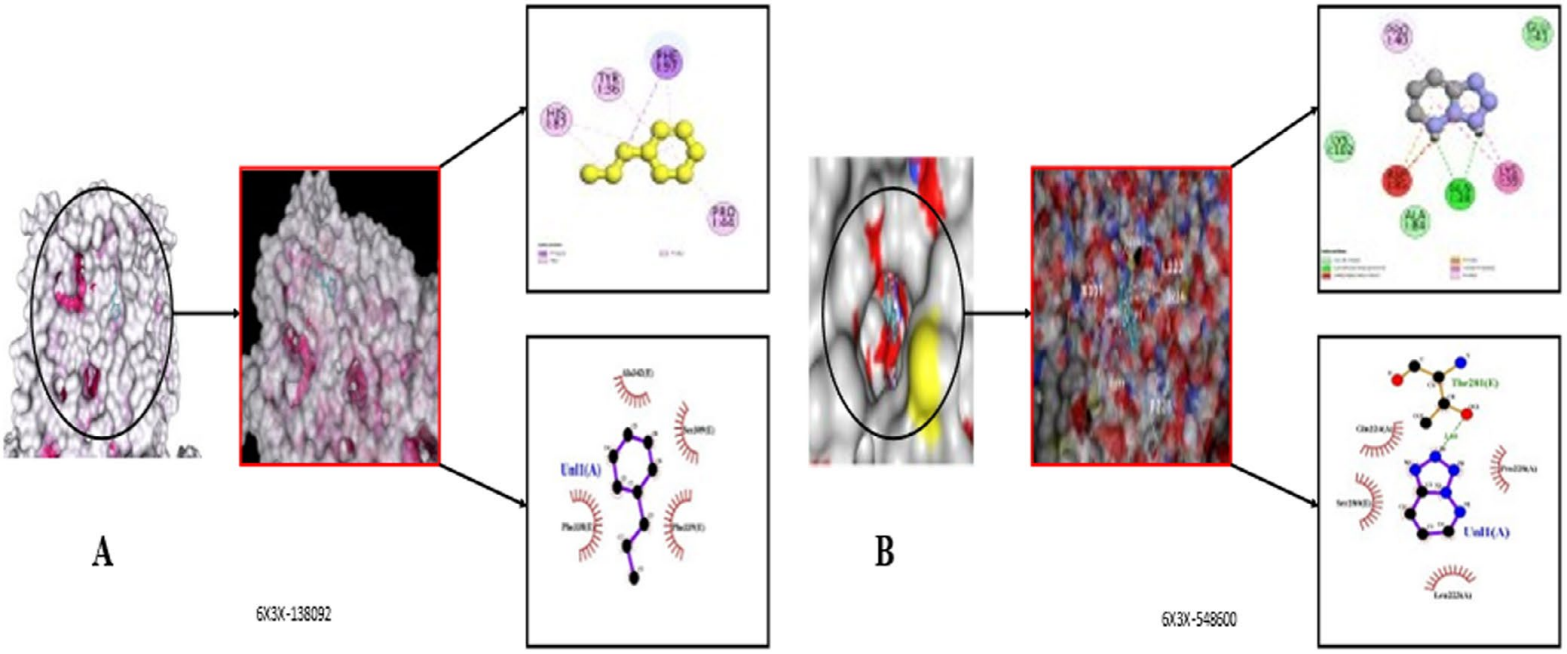
(A–B) Active‐site engagement of selected phytoconstituents—(A) Cyclohexene, 3‐propyl‐ and (B) Tetrazolo[1,5‐b]pyridazine—within the sedative target protein 6X3X.

The screened phytochemicals from *MECI* were subjected to in silico anxiolytic activity against protein target 2Z5X using both PyRx (AutoDock Vina) and CB‐Dock dockings. Docking results binding affinities and bonding interaction of amino acids residue with ligands were relatively the same using both tools are presented in the Table S2 and Table 14, respectively. Cyclohexene, 3‐propyl, 3‐Methyl‐2‐(2‐oxopropyl)furan and Tetrazolo[1,5‐b]pyridazine showed highest binding affinities (−7.7 to −7.2 kcal/mol) even more than the reference drug diazepam (−6.8 to −7.0 kcal/mol) are shown in Figure 11A–C, respectively. Methyl 12,13‐tetradecadienoate also had favorable interactions (−7.0 to −7.4 kcal/mol) is presented in Figure 12D. These findings suggest these phytochemicals have high affinity to bind with the anxiolytic target, justifying their candidacy for molecular dynamics simulations and future experimental validation.

**TABLE 14:** In silico binding affinity and non‐bonding interaction for anxiolytic activities, respectively, of the selected phytochemicals from *MECI*.

Compound name	Docking score (Kcal/mol)	Types of bonds	Amino acid residues
Cyclohexene, 3‐propyl	−7.7	Vander Waals	GLY A:110, THR A:205, GLU A:492, ASN A:125
		C‐H bond	ARG A:109, PHE A:112.
		Alkyl	ALA A: 111, TRP A: 128, TYR A: 124
		Pi‐Alkyl	HIS A:488
3‐Methyl‐2‐ (2‐oxopropyl)furan	−7.7	Vander Waals	ASN A:125, HIS A:488
		C‐H bond	GLU A:492
		Alkyl	ARG A:129
		Conventional Hydrozen Bond	THR A:205, ASP A:137, THR A:204
Tetrazolo[1,5‐b]pyridazine	−7.0	Vander Waals	LYS A:357, SER A:334, ILE A:326, GLU A:327
		Conventional Hydrozen Bond	GLU A:329, ASP A:328.
		Pi‐Alkyl	ARG A:172
Methyl 12,13‐tetradecadienoate	−7.0	Vander Waals	THR A:205, GLU A:492, ASN A:125, TYR A:124
		Pi‐Alkyl	HIS A: 488, TRP A: 128, PHE A: 112
Diazepam (standard drug)	−7.0	Vander Waals	GLY A:110, GLU A:492, HIS A:488, ASN A:125
		C‐H bond	GLU A:492
		Alkyl	TRP A:128, TYR A:124
		Conventional hydrozen bond	ASP A:137, THR A:204

#### 3.7.6 In Silico Evaluation of Sedative Activity

Molecular docking of selected phytochemicals from *MECI* against the sedative target 6X3X revealed strong binding interactions, corroborating the observed in vivo sedative effects. As summarized in Table S3 and Table 15, cyclohexene, 3‐propyl‐ (PubChem ID: 138092) and tetrazolo[1,5‐b]pyridazine (PubChem ID: 548600) exhibited the highest binding affinities in both PyRx (−8.6 and −8.1 kcal/mol) and CB‐Dock (−9.0 and −8.4 kcal/mol) platforms, surpassing the reference drugs Diazepam (−7.3/−7.8 kcal/mol) and Lorazepam (−7.4/−7.5 kcal/mol). Detailed non‐bonding interactions, including hydrogen bonds and hydrophobic contacts, are presented in Table 15, highlighting key residues responsible for stabilizing ligand–receptor complexes. The 3D binding pose of cyclohexene, 3‐propyl‐ within the active site of 6X3X is illustrated in Figure 12A, while tetrazolo[1,5‐b]pyridazine's engagement with the same target is depicted in Figure 12B, emphasizing hydrogen bonding, π–π stacking, and hydrophobic interactions. Collectively, these in silico findings demonstrate that *MECI* constituents have strong target‐binding candidates and provide a mechanistic rationale for their sedative activity, supporting further exploration as multi‐target CNS therapeutic agents.

**TABLE 15:** In silico binding affinity and non‐bonding interaction for sedative activities, respectively, of the selected phytochemicals from *MECI*.

Compound name	Docking score (Kcal/mol)	Types of bonds	Amino acid residues
Cyclohexene, 3‐propyl‐	−8.6	Pi‐Sigma	HIS I:87, TYR I:36
		Pi‐Alkyl	PRO I:44
		Alkyl	PHE I:97
Tetrazolo[1,5‐b]pyridazine	−8.1	Vander Waals	LYS I:102, ALA I:84
		Amide Pi‐Stacked	LYS I:39
		Pi‐Alkyl	PRO I:40
		Conventional Hydrozen Bond	GLN I:38
Diazepam (Standard)	−7.3	Vander Waals	THR A:205, GLU A:492, ASN A:125, TYR A:124
		Amide Pi‐Stacked	HIS A: 488, TRP A: 128, PHE A: 112
		Pi‐Alkyl	THR A:205, GLU A:492.
		Conventional Hydrozen Bond	HIS A: 488, TRP A: 128, PHE A: 112
Lorazepam (Standard)	−7.4	Vander Waals	PRO I:40
		Amide Pi‐Stacked	GLN I:38
		Pi‐Alkyl	LYS I:102, ALA I:84
		Pi‐Sigma	HIS I:87, TYR I:36

#### 3.7.7 In Silico Evaluation of Antidepressant Activity

**FIGURE 13:**
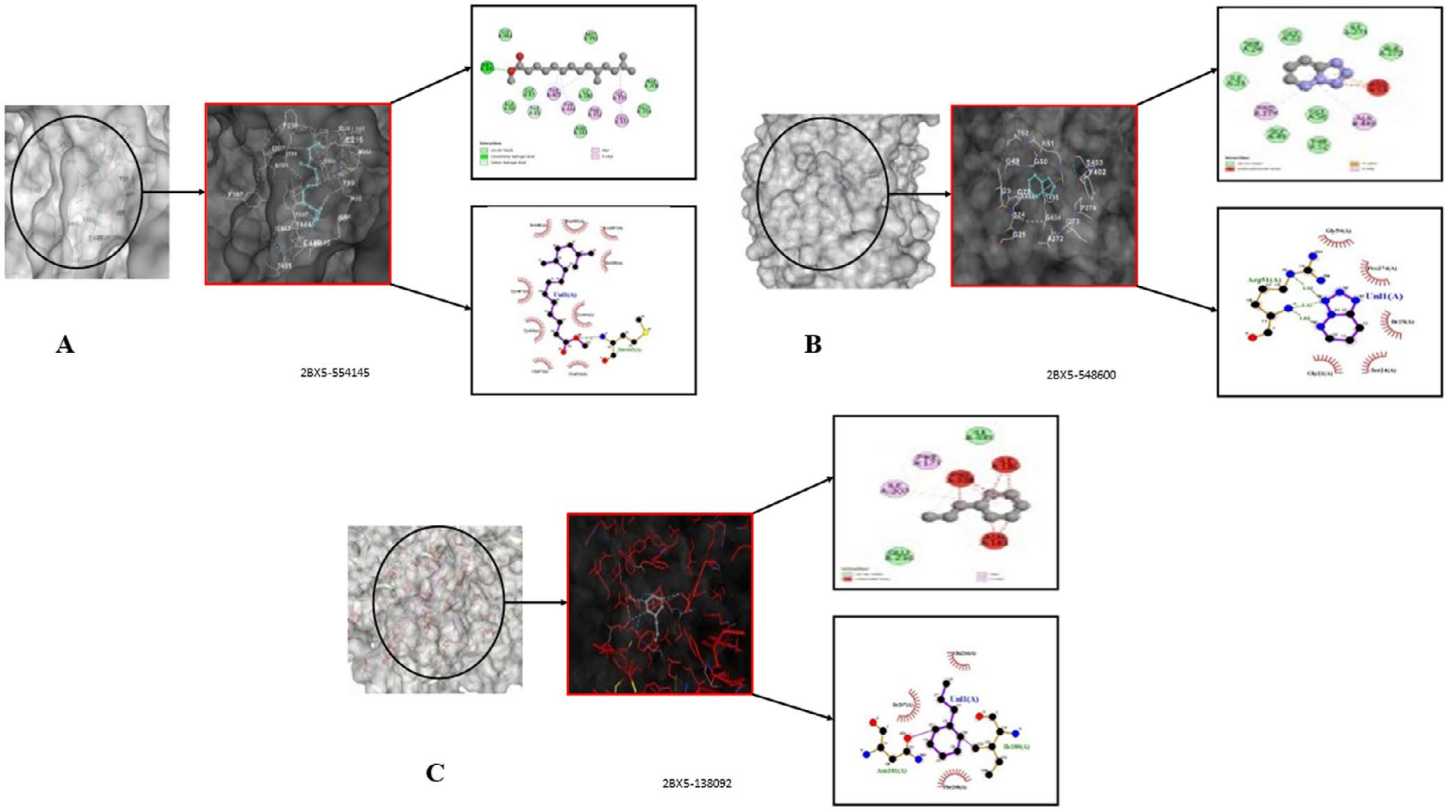
(A–C) Active‐site engagement of selected phytoconstituents—(A) Tetradecanoic acid, 10,13‐dimethyl‐, methyl ester, (B) Tetrazolo[1,5‐b]pyridazine, and (C) Cyclohexene, 3‐propyl—within the GABA receptor target protein 2BX5.

Molecular docking studies were performed on selected *MECI* phytochemicals against the antidepressant target protein (PDB ID: 2BX5) using PyRx and CB‐Dock, which revealed favorable binding affinities (Table S4). Among the screened compounds, methyl 10,13‐dimethyltetradecanoate (PubChem CID: 554145) demonstrated the highest binding energy (−8.5 and −8.3 kcal/mol) and was stably accommodated within the active site, as illustrated in Figure 13A. Tetrazolo[1,5‐b]pyridazine (PubChem CID: 548600) also exhibited significant affinity (−8.0 and −8.1 kcal/mol) with the target (Figure 13B). In contrast, 3‐propyl cyclohexene (PubChem CID: 138092) bound relatively weakly (−7.8 and −7.9 kcal/mol) (Figure 13C). The reference standard Fluoxetine (PubChem CID: 3380) displayed lower binding affinities (−7.6 and −7.4 kcal/mol), suggesting a higher binding energy of the screened phytochemicals compared to the standard. Detailed non‐bonding interactions, including hydrogen bonding, hydrophobic contacts, and π–π stacking, are summarized in Table 16, highlighting key residues responsible for stabilizing the ligand–receptor complexes. Collectively, these in silico findings indicate that *MECI* constituents possess strong binding interactions with the antidepressant target protein 2BX5, providing a mechanistic basis for the observed in vivo antidepressant effects and supporting their use as CNS therapeutic agents.

**TABLE 16:** In silico binding affinity and non‐bonding interactions of selected *MECI* phytochemicals with the antidepressant target protein 2BX5.

Compound name	Docking score (Kcal/mol)	Types of bonds	Amino acid residues
Tetradecanoic acid, 10, 13‐dimethyl‐, methyl ester.	−8.5	Vander waals	GLY A:443, MET A:350, ALA A:68, GLY A:67, GLU A:216
		Conventional Hydrozen Bond	MET A:445
		C‐H Bond	TRY A:69, ILE A:180
		Alkyl	ILE A:335, LEU A:337
		Pi‐Alkyl	TYR A:407, TYR A:444
Tetrazolo[1,5‐b]pyridazine	−8.1	Vander waals	SER A:24, GLY A:22, ILE A:73, ALA A:272
		Pi‐Alkyl	PRO A:274, ALA A:448
Cyclohexene, 3‐propyl	−7.9	Vander waals	GLU A:216, ILE A:335
		Alkyl	PHE A:177
		Pi‐Alkyl	ILE A:207
Fluoxetine	−7.6	Vander waals	ASN A:181, ILE A:180, GLU A:216, ALA A:272, THR A:52
		Alkyl	ILE A:3335, LEU A:337
		Pi‐Alkyl	ILE A:207

#### 3.7.8 Molecular Dynamic Simulation Analysis of Anxiolytic Activity

**FIGURE 14:**
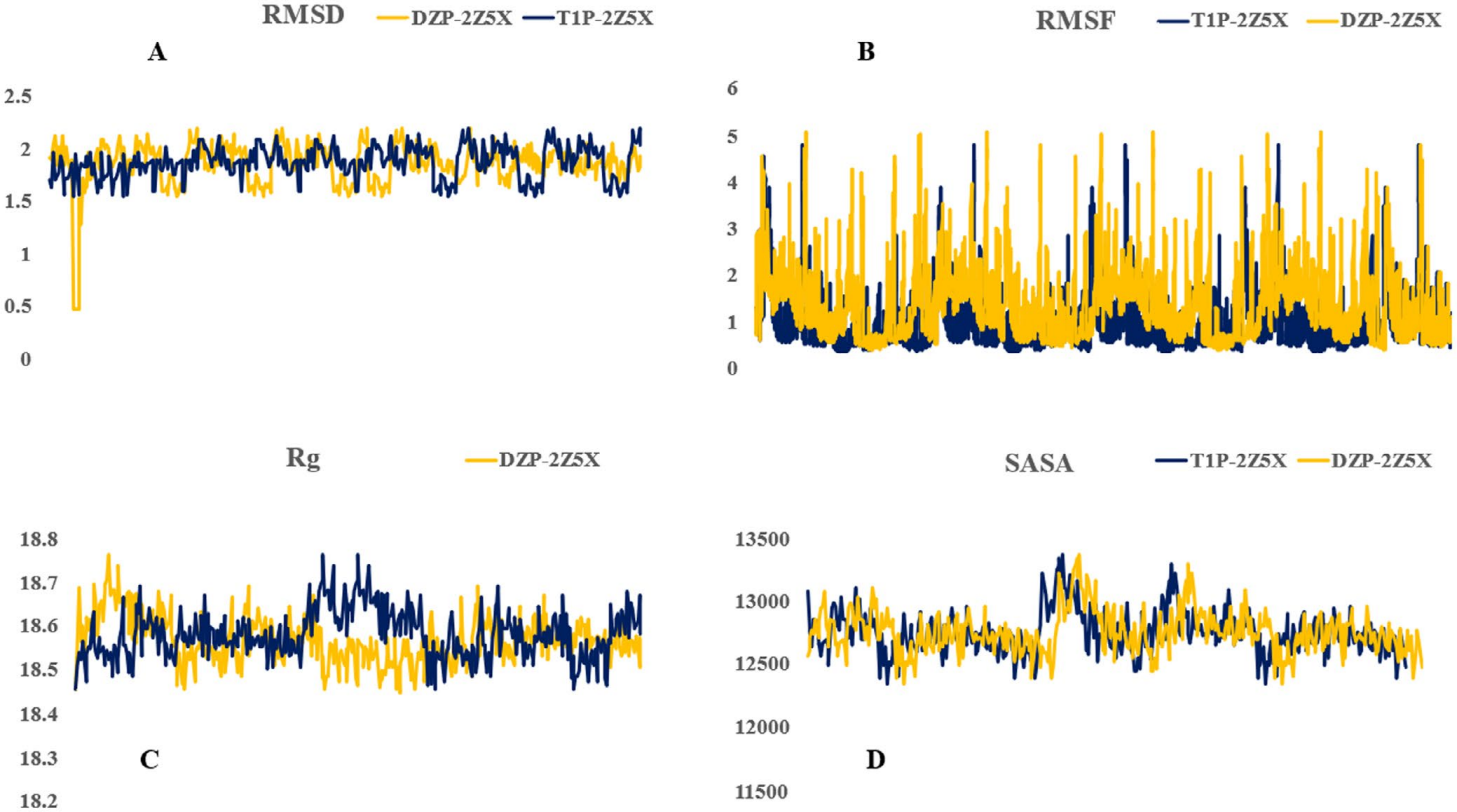
Molecular dynamics simulation analysis of top‐ranked anxiolytic complexes: (A) Root mean square deviation (RMSD) showing structural stability over simulation time; (B) root mean square fluctuation (RMSF) depicting residue‐level flexibility; (C) radius of gyration (Rg) indicating compactness of the complexes; (D) solvent accessible surface area (SASA) demonstrating exposure of protein surfaces. DZP‐275X (yellow) and T1P‐275X (blue) represent the two docked ligand–protein complexes analyzed over the simulation period.

Molecular dynamics simulations identified that the complex formed by 2Z5X and Tetrazolo[1,5‐b]pyridazine remained structurally stable over the course of a molecular dynamics simulation (Figure 14A–D). The RMSD plot (Figure 14A) was consistent and remained between 1.6–2.1 Å, further proposing no major conformational deviation, suggesting that ligand binding does not significantly affect the overall protein structure. Analysis of the root mean square fluctuation (RMSF; Figure 14B) suggested low residue‐level fluctuations (< 2 Å) for all systems, although local flexibility increased in and around loop regions, and more pronounced stabilization of the liganded system was apparent from comparably lower fluctuations upon binding. Additionally, the radius of gyration (Rg) plot (Figure 14C) revealed steady‐state levels (18.5–18.8 Å), indicative of structural compactness preservation, as well as slightly more stability in the ligand‐bound system. The SASA profile (Figure 14D) showed moderate fluctuations (≈12,500–13,200 Å^2^) with a small decrease in SASA in the ligand‐bound form, indicating a more compact and less solvent exposed conformation. In aggregate, these observations support that Tetrazolo[1,5‐b]pyridazine assembles a stable complex with 2Z5X, stabilizing structural architecture and limiting conformational movement—possibly helping to provide an avenue through which it exerts its anxiolytic‐like properties.

#### 3.7.9 Toxicity Analysis

The in silico toxicity profile of some principal bioactive compounds from *Combretum indicum* was found to be mostly favorable (Table 17). Non‐hepatotoxic and non‐cardiotoxic were the predictions of all compounds with respect to systemic organ‐specific adverse effects. Carcinogenicity was limited, with 3‐methyl‐2‐(2‐oxopropyl)furan and methyl 12,13‐tetradecadienoate showing activity in the low range; similarly, cytotoxicity predictions were generally negligible. Neurotoxicity was limited to tetrazolo[1,5‐b]pyridazine, consistent with selective CNS involvement seen for the expected pharmacological profile. Acute toxicity assessment as LD 50 showed low intrinsic acute toxicity, with long‐chain fatty acid esters and cyclohexene‐3‐propyl possessing LD 50 values of > 5000 mg/kg (Class 5), while those of heterocyclic compounds were slightly higher (Class 4). Correlatively, all these data underscore the promising preliminary toxicological profiles of *C. indicum* phytoconstituents and also support the translational merits of them as neuroactive leads having safe margin for further experimental investigation.

**TABLE 17:** Predicted toxicity profiles of selected *MECI* phytochemicals, including hepatotoxicity, cardiotoxicity, and mutagenicity.

Toxicity properties	Hepatotoxicity	Cardiotoxicity	Carcinogenicity	Cytotoxicity	Neurotoxicity	LD_50_	Toxicity Class
Tetrazolo[1, 5‐b]pyridazine	Inactive	Inactive	Inactive	Inactive	Active	1454 mg/kg	4
3‐Methyl‐2‐(2‐oxopropyl)furan	Inactive	Inactive	active (Mild)	Inactive	Inactive	1200 mg/kg	4
Cyclohexene,3‐propyl‐	Inactive	Inactive	Inactive	Inactive	Inactive	5000 mg/kg	5
Tetradecanoic acid, 10,13‐dimethyl‐, methyl ester	Inactive	Inactive	Inactive	Inactive	Inactive	5000 mg/kg	5
Methyl 12,13‐tetradecadienoate	Inactive	Inactive	active (Mild)	Inactive	Inactive	5000 mg/kg	5

## 4 Discussion

Small molecules from traditional medicine are the structurally diverse class of compounds that remain important leads for CNS‐active agents (Rahman et al. 2023). The methanolic extract of *Combretum indicum* *(MECI)* was characterized phytochemically and also investigated in a range of validated behavioral models to assess neuropharmacological properties with more translational significance using in silico methodologies to inform further study in this regard (Remali and Aizat 2024).

MECI induced dose‐dependent changes in mice that paralleled anxiolytic and antidepressant‐like activity in a range of behavioral assays. Elevated open‐arm exploration and reduced closed‐arm preference in the elevated plus maze (EPM) reflect decreased anxiety‐like behavior (Chandrashekar et al. 2017). Lower behavioral inhibition is also reflected in the increased time in the light compartment of the light/dark box and the increased head‐dipping in the hole‐board test. Importantly, locomotor activity was not significantly altered in the hole‐cross test in either the acute or chronic treatment paradigms, making nonspecific CNS depression an unlikely cause of this effect. Although these findings are consistent with anxiolytic‐like activity, they are merely behavioral correlates which do not provide direct support for any particular neurochemical pathways (Doukkali et al. 2015).

In addition, I significantly shortened the immobility period in the tail suspension test (TST) and forced swim test (FST), which are widely used screening models for antidepressant‐like effects (Rahman et al. 2023; Chandrashekar et al. 2017). However, it is important to view these results with caution. In addition to mood‐related behavioral changes, both FST and TST are sensitive to changes in locomotor activity, stress response, and coping styles. As such, drops in immobility are more than just an antidepressant effect; they may also be a consequence of greater behavioral activation or adaptive responses to stressors (Chen et al. 2015; Doukkali et al. 2015; Moniruzzaman et al. 2016).

The added value of FST for susceptibility to depression is its more specific behavioral relevance to depression, which when seen together with TST allows for a stronger behavioral evaluation (Ali and Engidawork 2022; Serefko et al. 2025). FST—often associated with energy‐conservation strategies—incorporates a hydric stressor and represents physiological acclimation to a hostile milieu. In contrast, the TST limits confounding factors such as hypothermia or capacity for swimming through the use of a suspension paradigm. When both tests are used, antidepressant‐like effects can be cross‐validated across different experimental paradigms; thus, providing greater confidence in the reproducibility of the behavioral findings (Nahar et al. 2026).

Phytochemical tests (GC–MS/MS and FTIR) indicated a chemically diverse profile among these groups, incorporating nitrogen‐containing heterocycles, oxygenated terpenoids, unsaturated fatty acids, as well as long‐chain esters. These identified compounds (e.g., tetrazolo[1,5‐b]pyridazine derivatives and fatty acid esters) have been described as CNS‐relevant bioactivities (Yuan et al. 2024). The present study, however, did not directly assess the contribution of individual compounds; nor did it examine possible synergistic or additive interactions within the extract.

Anxiety‐ and depression‐relevant protein targets that the selected phytoconstituents were predicted to bind to in silico with moderate to high confidence were also identified using PASS prediction. Moreover, molecular dynamics simulation results demonstrated that the ligand–protein complexes seem stable, with RMSD being steady, low RMSF, and stable radius of gyration (Rg). Although these findings further corroborate the above, they are not confirmatory, as none of the targets were subjected to experimental validation of target engagement (Shoaib et al. 2023; Azme et al. 2025).

A variety of compounds with predicted favorable intestinal absorption (*p* > 0.4) have also been identified, along with compounds with a logBB > 0, which are also good candidate CNS exposure (ADME profiling). While toxicity predictions and metabolic profiles were largely acceptable, these computational estimates of human biology need in vivo confirmation. Finally, while indicated behavioral effects as well as the literature on the neuropharmacology of escaped mice argue for the possible involvement of benzodiazepine‐sensitive but otherwise undetermined GABAergic pathways (Waddington 1978; Tohyama et al. 1991), such interpretations must be considered tentative until complemented by more selective receptor‐binding or neurochemical studies.

The behavioral effects described here, along with the identification of bioactive phytoconstituents and CNS bioavailability predicted therein, pose *C. indicum* as a probable source of neuroactive compounds from a translational standpoint. Nonetheless, the practical implications of these results are still basically limited. Additional studies to dissect active constituents, assess pharmacokinetics, ensure safety, and demonstrate efficacy will need to be conducted under more advanced preclinical and clinical conditions. We also need to further investigate the potential relevance of metabolism and more active metabolites in vivo.

## 5 Conclusion

This study demonstrates that the methanolic extract of *Combretum indicum (MECI)* has anxiolytic‐ and antidepressant‐like effects in preclinical behavioral models where sedative or locomotor impairments are not profound. Responses were dose‐dependent in multiple assays (suggesting potential neuropharmacological relevance). The phytochemical analysis indicated diverse constituents with the presence of terpenoids, heterocyclic compounds, and fatty acid derivatives, which may be responsible for the exhibited effects. In silico studies such as molecular docking, PASS prediction, ADMET profiling and toxicity predictions proposed potential multi‐target interactions and reasonable pharmacokinetic properties. In addition, the molecular dynamics simulation also provided evidence for Tetrazolo[1,5‐b]pyridazine in generating a stable complex with MAO‐A (PDB ID: 2Z5X), which allows potential notion as a lead compound. But these findings are preliminary and should be taken with caution. The active constituents remain isolated and have yet to be validated experimentally. Thus, more studies aimed at bioassay‐guided isolation, mechanistic validation, and clinical validation are necessary to establish the therapeutic goodness of *C. indicum* .

## 6 Limitations

This is a top‐notch result but there are several limitations to be addressed. In this study, we utilized a crude methanolic extract of *Combretum indicum* and did not isolate nor experimentally validate the exact bioactive compounds leading to the effects in question. Moreover, identification of compounds using GC–MS is tentative and spectral matching; hence, further confirmatory analysis is needed using advanced analytical techniques. All the behavioral assessments were limited to mice and do not adequately model human neuropsychiatric diseases. Furthermore, molecular docking and associated in silico analyses are predictive and not validated by concrete studies on receptor‐binding or specific biochemical pathways. However, important parameters like subchronic toxicity, pharmacodynamics, and sustained efficacy were not examined. The shortcomings mentioned above must be solved for future translational and clinical development.

## 7 Future Perspective

Future research efforts should focus on performing bioassay‐guided fractionation of *C. indicum* to identify and chemically characterize the most potent neuroactive component(s). Further mechanistic investigations, including electrophysiological and receptor‐subtype selective assays, will clarify the exact molecular targets. Pharmacodynamic studies and chronic in vivo models will be part of evaluating its long‐term efficacy and safety. More importantly, translational research work on human‐physiologic pharmacokinetic and CNS bioavailability will provide us the rational to develop *C. indicum* ‐derived phytotherapeutics as multi‐target agents for safe and efficacious control of anxiety and depression.

## Author Contributions

**Zobayed Islam:** conceptualization, investigation, writing – original draft, methodology, validation, writing – review and editing, visualization, software, formal analysis, data curation, resources. **Nilufar Sultana:** writing – original draft, writing – review and editing, methodology, software. **Md. Ashraful Alam:** methodology, formal analysis, software, writing – original draft. **S. M. Naim Uddin:** conceptualization, investigation, writing – original draft, writing – review and editing, software, formal analysis, supervision, project administration, resources, methodology. **Sabikun Naher:** conceptualization, investigation, writing – original draft, software. **Sakib Ahamed Bhuiyan:** methodology, writing – original draft, formal analysis, data curation.

## Funding

The authors have nothing to report. The study was carried out as part of the institutional academic curriculum in the University of Chittagong using available resources and infrastructures of the department.

## Ethics Statement

All experimental procedures involving animals were conducted in strict accordance with internationally accepted guidelines for the care and use of laboratory animals. The study protocol was reviewed and approved by the Animal Research Ethics Committee of the Faculty of Biological Sciences, University of Chittagong, Bangladesh (Approval No. AERB‐FBSCU‐20260220‐(2); approved on February 20, 2025). All efforts were made to minimize animal suffering and to reduce the number of animals used, in accordance with the principles of the 3Rs (Replacement, Reduction, and Refinement). Animals were handled by trained personnel, and appropriate measures, including anesthesia and predefined humane endpoints, were implemented to ensure welfare. At the end of the study, animals were humanely euthanized using an overdose of anesthetic administered via intraperitoneal injection, and death was confirmed prior to disposal.

## Supporting information


**Table S1:** Molecular docking scores of *MECI* constituents and reference drugs against CNS‐related targets using PyRx and CB‐Dock platforms (kcal/mol).
**Table S2:** In silico Docking Binding Score against 2Z5X for Anxiolytic activities, respectively, of the selected phytochemicals from *MECI*.
**Table S3:** In silico Docking Binding Score against 6X3X for Sedative activities, respectively, of the selected phytochemicals from *MECI*.
**Table S4:** In silico docking binding scores of selected *MECI* phytochemicals against 2BX5 for antidepressant activity (kcal/mol).

## Data Availability

The data generated and analyzed during this study are included in this published article and its Supporting Information files.
