# Supplementary material for: Phytochemical Characterization and Neuropharmacological Assessment of Combretum indicum Methanolic Extract With Integrated Molecular Docking and Dynamics Simulation for Anxiety and Depression Therapy
Source: Food Sci Nutr. 2026 May 23;14(5):e71918. doi: 10.1002/fsn3.71918 (PMC13240074; doi:10.1002/fsn3.71918)
Supplement: Supplementary file 1 — Table S1: Molecular docking scores of MECI constituents and reference drugs against CNS‐related targets using PyRx and CB‐Dock platforms (kcal/mol). Table S2: In silico Docking Binding Score against 2Z5X for Anxiolytic activities, respectively, of the selected phytochemicals from MECI. Table S3: In silico Docking Binding Score against 6X3X for Sedative activities, respectively, of the selected phytochemicals from MECI. Table S4: In silico docking binding scores of selected MECI phytochemicals against 2BX5 for antidepressant activity (kcal/mol). [file FSN3-14-e71918-s001.docx]

**Supplementary Table-01:** Molecular docking scores of *MECI* constituents and reference drugs against CNS-related targets using PyRx and CB-Dock platforms (kcal/mol).

| PID | Sedative | | Anxiolytic | | Anti-depressant | |
| --- | --- | --- | --- | --- | --- | --- |
|  | 6X3X(PYRX) | 6X3X(CB-DOCK) | 2Z5X(PYRX) | 2Z5X(CB-DOCK) | 2BX5(PYRX) | 2BX5(CB-DOCK) |
| 118118 | -4.2 | -3.5 | -4.4 | -6.1 | -5.9 | -5.6 |
| 12587 | -3.8 | -3.5 | -4.7 | -5.6 | -6.7 | -5.5 |
| 12600623 | -4 | -4.6 | -4.7 | -5.3 | -6.1 | -5.5 |
| 138092 | -8.6 | -9.0 | -7.7 | -7.5 | -7.8 | -7.9 |
| 17469 | -3.1 | -4.4 | -5 | -5.6 | -6.3 | -5.2 |
| 175468 | -4.3 | -4.3 | -5.1 | -5.3 | -6.1 | -5.2 |
| 176505 | -4.4 | -4.2 | -4.1 | -5.3 | -6 | -5.2 |
| 219522 | -3.3 | -4.2 | -5.3 | -5.2 | -6.3 | -5.1 |
| 2969 | -4.3 | -4.2 | -7 | -5.2 | -6.2 | -5 |
| 5280435 | -5 | -3.4 | -3.8 |  | -6.2 | -6.2 |
| 545772 | -4 | -3.4 | -7.7 | -7.5 | -6.1 | -6.2 |
| 548381 | -2.7 | -3.4 | -5 | -4.5 | -6 | -6.1 |
| 548600 | -8.1 | -8.4 | -7.7 | -7.2 | -8.0 | -8.1 |
| 549927 | -4.1 | -4.6 | -5 | -5.1 | -6.3 | -6 |
| 554101 | -4.3 | -4.4 | -5 | -5 | -6.5 | -5.6 |
| 554101 | -3.6 | -4.3 | -6.4 | -4.9 | -5 | -5.5 |
| 554145 | -4.6 | -4.2 | -4.6 | -4.8 | -8.5 | -8.3 |
| 557163 | -5.3 | -4.2 | -4.7 | -7 | -5.9 | -5.7 |
| 560094 | -3.3 | -4.2 | -4.4 | -6.8 | -5.2 | -5.5 |
| 568126 | -3.9 | -4.1 | -7 | -6.8 | -5.7 | -5.3 |
| 568888 | -4.3 | -3.7 | -5.3 | -6.7 | -5.5 | -5.2 |
| 6514857 | -5.6 | -4.3 | -5.7 | -6.7 | -5.6 | -5.2 |
| 71684441 | -4.9 | -4.3 | -4.5 | -6 | -5.5 | -5.2 |
| 76255 | -4.7 | -5.3 | -4.2 | -5.8 | -5.1 | -5.1 |
| 85984 | -3.7 | -5.2 | -4.1 | -7 | -5.9 | -5 |
| 91692385 | -4.3 | -5.1 | -7.0 | -7.4 | -6.7 | -5 |
| 91692472 | -4.6 | -5 | -4.6 | -6.1 | -6.1 | -6.5 |
| 9548 | -4.3 | -4.3 | -4.4 | -5.6 | -5.7 | -6.5 |
| Floxetine(3386) | - | - | - | - | -7.6 | -7.4 |
| 3016(Diazepam) | - | - | -6.8 | -7.0 | -6 | -5 |
| 3958(Lorazepam) | −7.4 | −7.5 | - | - | - | - |

**Supplementary Table-02**: In silico Docking Binding Score **against 2Z5X** for Anxiolytic activities, respectively, of the selected phytochemicals from *MECI*.

| Compound Name | PubChem ID | Binding Affinity (Tool-PYRX, kcal/mol) | Binding Affinity (Tool-CB DOCK, kcal/mol) |
| --- | --- | --- | --- |
| Cyclohexene, 3-propyl | 138092 | −7.7 | −7.5 |
| 3-Methyl-2-(2-oxopropyl)furan | 545772 | −7.7 | −7.5 |
| Tetrazolo[1,5-b]pyridazine | 548600 | −7.7 | −7.2 |
| Methyl 12,13-tetradecadienoate | 91692385 | −7.0 | −7.4 |
| Diazepam (reference drug) | 3016 | −6.8 | −7.0 |

**Supplementary Table-03:** In silico Docking Binding Score **against 6X3X** for Sedative activities, respectively, of the selected phytochemicals from *MECI*.

| Compound Name | Compound (PubChem ID) | Binding Affinity (PyRx) (kcal/mol) | Binding Affinity (CB-Dock) (kcal/mol) |
| --- | --- | --- | --- |
| cyclohexene, 3-propyl- | 138092 | −8.6 | −9.0 |
| tetrazolo[1,5-b]pyridazine | 548600 | −8.1 | −8.4 |
| Diazepam (Standard) | 3016 | −7.3 | −7.8 |
| Lorazepam (Standard) | 3958 | −7.4 | −7.5 |

**Supplementary Table-04:** In silico docking binding scores of selected *MECI* phytochemicals against 2BX5 for antidepressant activity (kcal/mol).

| Compound Name | PubChem ID | Binding Affinity (Tool-PYRX, kcal/mol) | Binding Affinity (Tool-CB DOCK, kcal/mol) |
| --- | --- | --- | --- |
| Tetradecanoic acid,10,13-dimethyl-,methyl ester. | 554145 | -8.5 | -8.3 |
| Tetrazolo[1,5-b]pyridazine | 548600 | -8.0 | -.8.1 |
| Cyclohexene, 3-propyl | 138092 | -7.8 | -.7.9 |
| Fluoxetine(Standard drug) | 3386 | -7.6 | -7.4 |
